# Elimination of the four extracellular matrix molecules tenascin-C, tenascin-R, brevican and neurocan alters the ratio of excitatory and inhibitory synapses

**DOI:** 10.1038/s41598-019-50404-9

**Published:** 2019-09-26

**Authors:** Christine Gottschling, David Wegrzyn, Bernd Denecke, Andreas Faissner

**Affiliations:** 1 Department of Cell Morphology and Molecular Neurobiology, Ruhr University Bochum, Universitaetsstr. 150, building NDEF 05, D-44801, Bochum, Germany; 2 IZKF Aachen, RWTH Aachen, D-52074 Aachen, Germany

**Keywords:** Synaptic development, Neural circuits, Extracellular signalling molecules

## Abstract

The synaptic transmission in the mammalian brain is not limited to the interplay between the pre- and the postsynapse of neurons, but involves also astrocytes as well as extracellular matrix (ECM) molecules. Glycoproteins, proteoglycans and hyaluronic acid of the ECM pervade the pericellular environment and condense to special superstructures termed perineuronal nets (PNN) that surround a subpopulation of CNS neurons. The present study focuses on the analysis of PNNs in a quadruple knockout mouse deficient for the ECM molecules tenascin-C (TnC), tenascin-R (TnR), neurocan and brevican. Here, we analysed the proportion of excitatory and inhibitory synapses and performed electrophysiological recordings of the spontaneous neuronal network activity of hippocampal neurons *in vitro*. While we found an increase in the number of excitatory synaptic molecules in the quadruple knockout cultures, the number of inhibitory synaptic molecules was significantly reduced. This observation was complemented with an enhancement of the neuronal network activity level. The *in vivo* analysis of PNNs in the hippocampus of the quadruple knockout mouse revealed a reduction of PNN size and complexity in the CA2 region. In addition, a microarray analysis of the postnatal day (P) 21 hippocampus was performed unravelling an altered gene expression in the quadruple knockout hippocampus.

## Introduction

The functionality of neuronal synapses is warranted by the correspondence of a pre- and a postsynapse that form the synaptic cleft which allows for the transmission of an electrical activity by chemical neurotransmitters. Nowadays, it is obvious that glia cells such as astrocytes^1–4^ as well as extracellular matrix (ECM) molecules^5–9^ contribute to the process of synaptic information transfer. Most of the ECM molecules in the brain are produced and released by astrocytes and neurons themselves^10^. One specialized structure of the ECM is termed perineuronal net (PNN) and was originally discovered by Camillo Golgi more than a century ago^11^. PNNs surround inhibitory fast spiking parvalbumin-positive GABAergic interneurons^12–14^, but are also associated with further subpopulations, for example excitatory pyramidal neurons in the cerebral cortex^15,16^. The core structure of PNNs consists of chondroitin sulfate proteoglycans (CSPGs) of the lectican family^17,18^, hyaluronan^19,20^, tenascins, particularly tenascin-R (TnR)^21,22^ and link proteins^23,24^ for stabilization. When the composition of PNNs is impaired, the neuronal synaptic plasticity in form of LTP is affected, as has been reported for the tenascin-C (TnC)^7^, TnR^25^, brevican^26^ and neurocan^27^ knockout mice. In the present study, the interplay between neurons and astrocytes in conjunction with the PNN structure was analysed using the quadruple knockout mouse, which lacks the four ECM components TnC, TnR, neurocan and brevican. The first study describing this knockout mouse revealed an upregulation of the proteins fibulin-1 and fibulin-2 in the brains of one month old mice^28^. In a previous study our laboratory investigated hippocampal neurons of the quadruple knockout mouse *in vitro* and found diminished PNNs and an altered expression of synaptic proteins that were accompanied by a decreased frequency of mEPSCs and mIPSCs after 14 and 21 DIV^29^. This also translates in an altered synaptic plasticity *in vivo* as the quadruple knockout mouse exhibited a reduced short-term depression connected with changed frequency dependence in the dentate gyrus of the hippocampus subjected to a classical LTP paradigm^30^.

In the light of these observations we sought to expand our insight into the importance of the ECM for the process of synaptogenesis. To this end we explored the quadruple knockout mouse in conjunction with the indirect co-culture system of cortical astrocytes and hippocampal neurons^29,31^. We examined the proportion of inhibitory and excitatory synapses in culture, as well as the spontaneous activity of the neuronal networks using the multielectrode array (MEA) approach^32^. We also monitored the formation of PNNs in the developing hippocampus of quadruple knockout mice *in vivo*, as it has recently been reported that PNNs are highly enriched in the CA2 region of the murine hippocampus^33^. Moreover, we compared the gene expression in this brain structure at postnatal day (P) 21 in wildtype and quadruple knockout mice by microarray analysis.

## Results

### Enhancement of excitatory synapses in the quadruple knockout mouse

**Figure 1:**
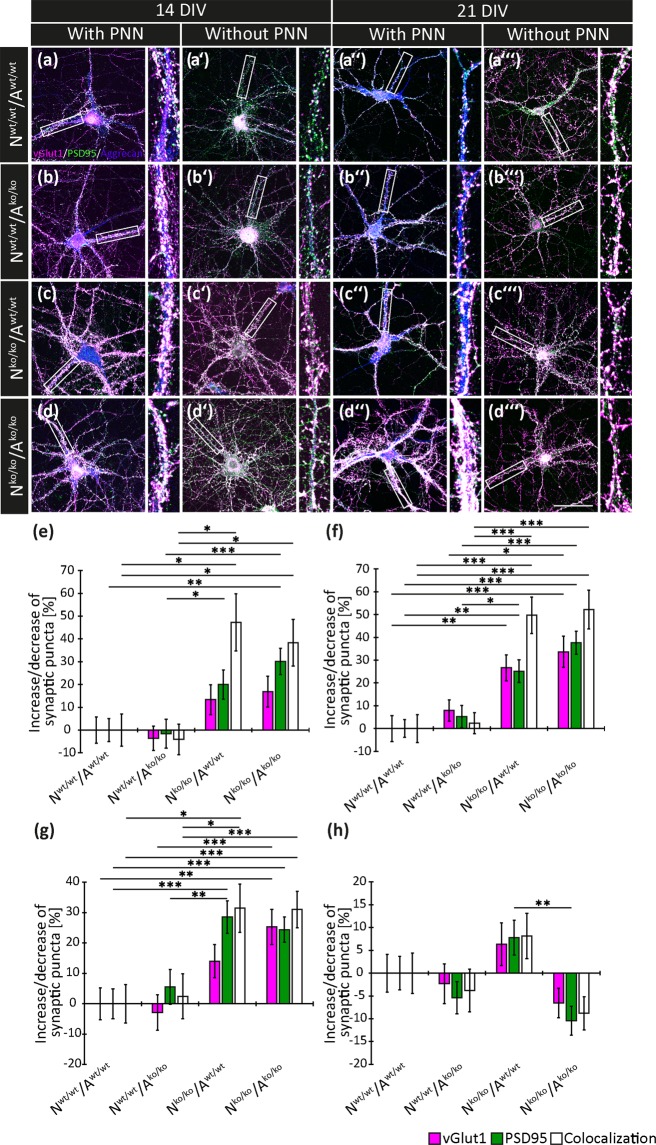
Expression of the synaptic puncta vGlut1 and PSD95 in hippocampal neurons after 14 and 21 DIV. (**a**,**a’**,**a”**,**a”’**,**b**,**b’**,**b”**,**b”’**,**c**,**c’**,**c”**,**c”’**,**d**,**d’**,**d”**,**d”’**) Representative images of the immunocytochemical stainings of the presynaptic protein vGlut1 (puncta appearing in magenta) and the postsynaptic protein PSD95 (green puncta) after 14 DIV and 21 DIV for the four conditions (N^wt/wt^/A^wt/wt^, N^wt/wt^/A^ko/ko^, N^ko/ko^/A^wt/wt^, N^ko/ko^/A^ko/ko^) are shown. The white puncta reflect the colocalization of both proteins and indicate structural synapses. The PNNs were visualized using aggrecan (blue), which is visible in (**a**,**a”**,**b**,**b”**,**c**,**c”**,**d**,**d”**) for PNN-bearing neurons. In (**a’**,**a”’**,**b’**,**b”’**,**c’**,**c”’**,**d’**,**d”’**), neurons devoid of PNNs are exemplified. Next to each of the micrographs is a higher magnification of one exemplary neurite (white box in each image) showing the individual synaptic puncta in more detail. Scale bar: 50 µm in D”’. (**e**,**f**,**g**,**h**) Analysis of the relative increase or decrease of the number of synaptic puncta in percent after 14 (**e**,**f**) and 21 DIV (**g**,**h**). PNN-wearing knockout neurons after 14 DIV (**e**) combined with wildtype astrocytes (N^ko/ko^/A^wt/wt^) and knockout astrocytes (N^ko/ko^/A^ko/ko^) showed a significant rise of excitatory synapses. The same applies for PNN-free neurons after 14 DIV (**f**). PNN-wearing knockout neurons (**g**) co-cultivated with knockout astrocytes (N^ko/ko^/A^ko/ko^) showed a significant enhancement of structural synapses in culture compared to the control (N^wt/wt^/A^wt/wt^) after 21 DIV. No significant differences were revealed after 21 DIV for the number of synaptic puncta of PNN-negative knockout neurons (**h**) compared to the control condition. Statistics: Five independent experiments (biological replicates N = 5) were performed choosing randomly 20 neurons with (n = 20) and 20 neurons without PNNs (n = 20) per each condition. In sum, 800 neurons were analysed. Data are expressed as mean ± SEM (ANOVA and Scheffé post hoc test for PSD-95 data sets and Kruskal-Wallis test for vGlut1 and Colocalization data sets, p ≤ 0.05).

Hippocampal neurons and cortical astrocytes of wildtype and quadruple knockout mice were cultivated in the four possible combinations (N^wt/wt^/A^wt/wt^, N^wt/wt^/A^k^°^/k^°, N^k^°^/ko^/A^wt/wt^, N^ko/ko^/A^ko/ko^), in the following referred to as conditions. For the detection of excitatory synapses after 14 DIV and 21 DIV (Fig. 1), the presynaptic protein vGlut1 and the postsynaptic protein PSD95 were examined using specific antibodies. We consider situations where pre- and postsynaptic puncta are in register and the corresponding fluorescence signals overlap as structural synapses^31,34^. In contrast puncta that are not colocalized with their corresponding partner are designated as delocalized, for the purpose of this study. To distinguish between PNN wearing (Fig. 1a–d+a”-d”) and PNN-free neurons (Fig. 1a’–d’+a”’–d”’), aggrecan, a major constituent of the PNN structure, was used^35^. In case of the formation of structural synapses white puncta indicating the overlap of pre- and postsynaptic puncta were detectable.

Knockout neurons cultivated in the presence of knockout astrocytes (N^ko/ko^/A^ko/ko^), which were surrounded by PNNs showed a significant upregulation of PSD95 and colocalized vGlut1 puncta of 30.18 ± 5.79% (p = 0.002) and 38.31 ± 10.19% (p = 0.040) compared to the control (N^wt/wt^/A^wt/wt^) after 14 DIV (Fig. 1e). The delocalized vGlut1 puncta were not significantly increased (16.90 ± 6.78%, p = 0.091). No significant shift of excitatory puncta was observable for knockout neurons grown with wildtype astrocyte (N^ko/ko^/A^wt/wt^) where vGlut1 and PSD95 puncta shifted by 13.38 ± 6.55% (p = 0.185) and 19.97 ± 6.47% (p = 0.392), respectively. A significant enhancement of 47.29 ± 12.49% (p = 0.050) could be detected for excitatory synapses. Hence, the astrocytes seemed not capable to rescue the neuronal phenotype. When wildtype neurons were cultivated together with knockout astrocytes (N^wt/wt^/A^ko/ko^), no alteration of the number of synaptic puncta was detectable, (vGlut1 puncta: −3.56 ± 5.30% (p = 0.472), PSD95 puncta: −1.62 ± 6.37% (p = 1.000), colocalized puncta: −4.06 ± 6.73% (p = 1.000), Fig. 1, Supplementary Table S1a,b), suggesting that the distinct genotypes of the astrocytes did not intervene in the formation of excitatory synapses.

An even larger rise of excitatory structural synapses was determined for PNN-free knockout neurons cultivated with knockout astrocytes (N^ko/ko^/A^ko/ko^) (Fig. 1f). Here, an increase was measured of 52.24 ± 8.51% (p < 0.001) compared to the control condition (N^wt/wt^/A^wt/wt^). The delocalized vGlut1 and PSD95 puncta were also significantly elevated by 33.69 ± 6.82% (p < 0.001) and 37.64 ± 5.05% (p < 0.001). Analogous results were obtained for knockout neurons grown in the presence of wildtype astrocytes (N^ko/ko^/A^wt/wt^) where the vGlut1 puncta were significantly increased by 26.65 ± 5.65% (p = 0.002), as well as the PSD95 puncta (25.14 ± 4.98%, p = 0.002) and the excitatory synapses (49.73 ± 8.01%, p < 0.001). In contrast, when wildtype neurons were co-cultivated with knockout astrocytes (N^wt/wt^/A^ko/ko^), no differences with regard to excitatory synapses were measured (vGlut1: 7.94 ± 4.71% (p = 0.505), PSD95: 5.28 ± 4.92% (p = 1.000), structural synapses: 2.33 ± 4.62% (p = 1.000), Fig. 1f, Supplementary Table S1a,b).

After 21 DIV, PNN wearing knockout neurons co-cultivated with knockout astrocytes (N^ko/ko^/A^ko/ko^) (Fig. 1g) also exhibited a strong increase of excitatory synaptic puncta (structural synapses: 31.03 ± 5.9%, p < 0.001; delocalized vGlut1 puncta: 25.29 ± 5.76%, p = 0.008; delocalized PSD95 puncta: 24.38 ± 4.14%, p = 0.001). Knockout neurons combined with wildtype astrocytes (N^ko/ko^/A^wt/wt^) displayed a higher formation of excitatory synapses (PSD95 puncta: 28.56 ± 5.34%, p = 0.001; structural synapses: 31.43 ± 7.91%, p = 0.024). In contrast, the delocalized vGlut1 puncta were not significantly altered (14.03 ± 5.50%, p = 0.618). Wildtype neurons co-cultivated with knockout astrocytes (N^wt/wt^/A^ko/ko^) showed no change in their number of synaptic puncta (vGlut1: −2.86 ± 5.83%, p = 1.000; PSD95: 5.59 ± 5.72%, p = 1.000; colocalized puncta: 2.43 ± 7.41%, p = 1.000). In the absence of PNNs, the only significant difference that was observed concerned the number of PSD95 puncta which differed between knockout neuron combinations N^ko/ko^/A^ko/ko^ compared to N^ko/ko^/A^wt/wt^ (p = 0.005) (Fig. 1h).

In conclusion, the elimination of the four extracellular matrix molecules TnC, TnR, brevican and neurocan resulted in a remarkable increase of excitatory synapses in PNN-positive as well as PNN-negative mutant neurons after 14 DIV.

### Reduction of inhibitory synapses in the quadruple knockout mouse

**Figure 2:**
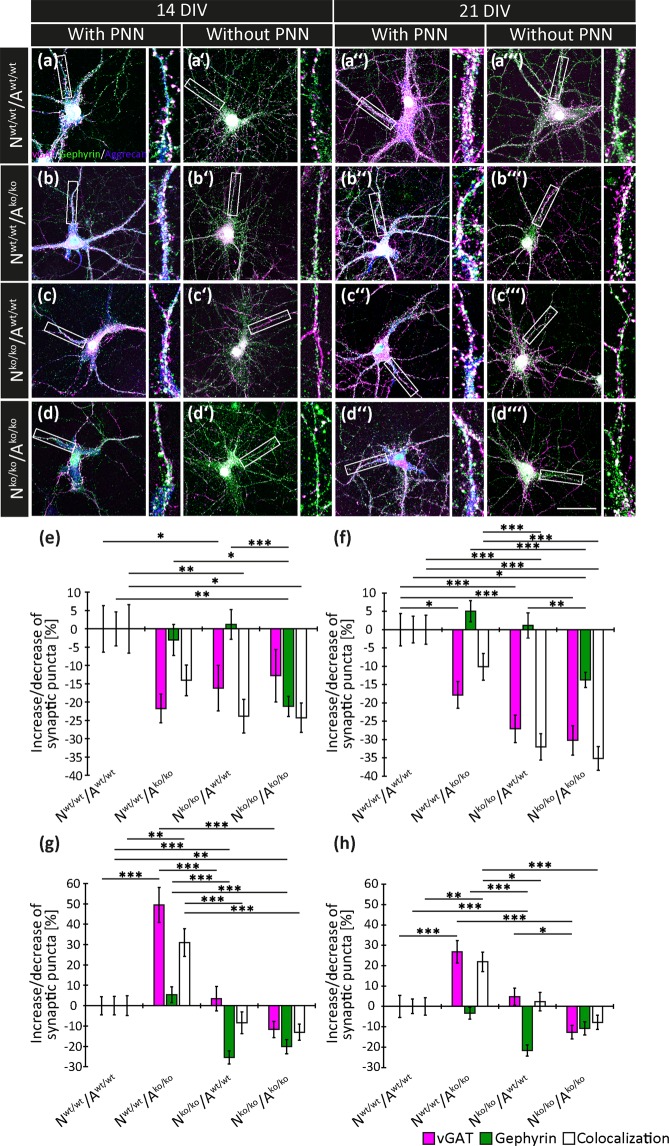
Expression of the synaptic puncta vGAT and gephyrin in hippocampal neurons after 14 and 21 DIV. (**a**,**a’**,**a”**,**a”’**,**b**,**b’**,**b”**,**b”’**,**c**,**c’**,**c”**,**c”’**,**d**,**d’**,**d”**,**d”’**) Representative images show the immunocytochemical stainings of hippocampal neurons after 14 and 21 DIV with the presynaptic marker vGAT (magenta puncta) and the postsynaptic marker gephyrin (green puncta) to detect inhibitory synapses in all four conditions (N^wt/wt^/A^wt/wt^, N^wt/wt^/A^ko/ko^, N^ko/ko^/A^wt/wt^, N^ko/ko^/A^ko/ko^). Colocalization of these proteins leads to the appearance of white puncta, which we define as structural inhibitory synapses in the neuronal cultures. To distinguish between neurons with (**a**,**a”**,**b**,**b”**,**c**,**c”**,**d**,**d”**) and without PNNs (**a’**,**a”’**,**b’**,**b”’**,**c’**,**c”’**,**d’**,**d”’**), aggrecan (blue) was used to visualize the PNNs. The higher magnification next to each image (white box in each image) displays one representative neurite to allow for a more detailed view on the distribution of the synaptic puncta. Scale bar: 50 µm in d”’. (**e**,**f**,**g**,**h**) The total number of the vGAT, gephyrin and colocalized puncta was quantified and the percentage of the increase/decrease of the number of synaptic puncta was thus determined after 14 (**e**,**f**) and 21 DIV (**g**,**h**). Irrespective of whether the neurons were covered by PNN (**e**) or not (**f**), the knockout neurons showed a significantly reduced number of synaptic puncta. PNN-wearing knockout neurons co-cultivated with knockout astrocytes for 21 DIV (N^ko/ko^/A^ko/ko^) exhibited a significant lower number of gephyrin puncta in comparison to the control (N^wt/wt^/A^wt/wt^) (**g**), whereas the number of structural synapses was not significantly altered. Knockout neurons combined with knockout astrocytes and without PNNs showed no significant change in the number of their synaptic puncta (**h**). Statistics: Five independent experiments (N = 5) were performed choosing randomly 20 neurons with (n = 20) and 20 neurons without PNNs (n = 20) per each condition. In sum, 800 neurons were analysed. Data are expressed as mean ± SEM (Kruskal-Wallis test, p ≤ 0.05).

For the detection of inhibitory synapses, vGAT was used as a presynaptic and gephyrin as a postsynaptic marker^36^ after 14 and 21 DIV (Fig. 2a,a”,b,b”,c,c”,d,d”). Compared to the control (N^wt/wt^/A^wt/wt^) and different from the situation of excitatory synapses described above, the number of structural inhibitory synapses appeared reduced in all conditions involving mutant cells, irrespective of the presence or absence of PNNs (Fig. 2 and Supplementary Table S2a,b). For PNN-positive knockout neurons co-cultivated with knockout astrocytes for 14 DIV (N^ko/ko^/A^ko/ko^), the gephyrin puncta as well as the inhibitory synapses were significantly reduced (Fig. 2e) by −21.15 ± 2.75% (p = 0.008) and −24.25 ± 3.98% (p = 0.016). The vGAT puncta were not affected (−12.79 ± 7.15%, p = 0.062). Analogous results were obtained for PNN-positive knockout neurons grown with wildtype astrocytes (N^ko/ko^/A^wt/wt^) where inhibitory synapses diminished by −23.85 ± 3.98% (p = 0.007). In contrast, the vGAT and gephyrin puncta were not significantly modified, −16.18 ± 6.23%, (p = 0.028) and 1.23 ± 4.05% (p = 1.000), respectively. No significant alteration of synaptic puncta was visible in PNN-positive wildtype neurons co-cultivated with knockout astrocytes (Supplementary Table S2a,b).

When knockout neurons not surrounded by a PNN were cultivated for 14 DIV (Fig. 2f), in the presence of knockout astrocytes (N^ko/ko^/A^ko/ko^), the cultures presented a significant decrease of synaptic puncta compared to the control (N^wt/wt^/A^wt/wt^) which amounted to −30.22 ± 4.01% (p < 0.001) for vGAT puncta, −13.70 ± 2.08% (p = 0.049) for gephyrin puncta and −35.11 ± 3.24% (p < 0.001) for structural inhibitory synapses. A significant reduction of vGAT puncta by −27.09 ± 3.78% (p < 0.001) and inhibitory synapses by −32.01 ± 3.64% (p < 0.001) could furthermore be identified on knockout neurons grown in the presence of wildtype astrocytes (N^ko/ko^/A^wt/wt^). Interestingly, the percentage of gephyrin puncta appeared unchanged in this condition (1.15 ± 3.45%, p = 1.000). The combination of wildtype neurons with knockout astrocytes (N^wt/wt^/A^ko/ko^) resulted in a decrease of vGAT puncta by −17.83 ± 3.64% (p = 0.035), while the gephyrin puncta as well as the inhibitory synapses remained unaffected (4.99 ± 2.88%, p = 0.372; and −10.12 ± 3.64%, p = 0.667). Thus, after 14 DIV the fraction of inhibitory synapses was reduced both on PNN-positive and PNN-negative knockout neurons.

An analogous analysis was performed after 21 DIV (Fig. 2g,h, Supplementary Table S2a,b). Under this schedule, PNN-positive knockout neurons grown with knockout astrocytes (N^ko/ko^/A^ko/ko^) developed significantly lesser gephyrin puncta, while the vGAT puncta and inhibitory synapses were not altered compared to the control (N^wt/wt^/A^wt/wt^); vGAT: −11.66 ± 4.01% (p = 0.287), gephyrin: −20.15 ± 3.50% (p = 0.004) and colocalized puncta: −13.01 ± 3.96% (p = 0.354). The same was examined for the synaptic puncta of knockout neurons with wildtype astrocytes (N^ko/ko^/A^wt/wt^). Here, a significant reduction of −25.44 ± 3.24% (p < 0.001) of gephyrin puncta was quantified, whereas the vGAT (3.45 ± 5.95%, p = 1.000) and the structural synapses (−8.40 ± 5.35%, p = 0.396) remained unchanged. Interestingly, wildtype neurons with PNNs co-cultured with knockout astrocytes (N^wt/wt^/A^ko/ko^) exhibited a significant enhancement of the vGAT puncta by 49.47 ± 8.53% (p < 0.001) and inhibitory synapses by 30.97 ± 6.82% (p = 0.005). The gephyrin puncta were not changed in this condition (5.42 ± 3.92% (p = 0.807)).

Neurons without a PNN (Fig. 2h) showed the following expression of synaptic puncta in comparison to the control (N^wt/wt^/A^wt/wt^). No alteration in the percentage increase/decrease of synaptic puncta was found for knockout neurons grown with knockout astrocytes (N^ko/ko^/A^ko/ko^) (vGAT: −12.73 ± 3.33% (p = 0.879), gephyrin: −10.77 ± 3.20% (p = 0.098) and colocalized puncta: −7.83 ± 3.45% (p = 1.000). The gephyrin puncta were significantly reduced, by −21.63 ± 2.76% (p < 0.001), when knockout neurons were co-cultivated with wildtype astrocytes (N^ko/ko^/A^wt/wt^), but no change could be found for the vGAT and inhibitory synapses in this condition (vGAT: 4.80 ± 4.22% (p = 0.795) and colocalized puncta: 2.38 ± 4.52% (p = 1.000)). Like before seen for neurons with PNNs (Fig. 2g), wildtype neurons combined with knockout astrocytes (N^wt/wt^/A^ko/ko^) exhibited a significantly enhanced percentage of vGAT puncta by 26.78 ± 5.53% (p < 0.001) and of inhibitory synapses by 21.93 ± 4,79% (p = 0.007), whereas the percentage of gephyrin puncta was not modified (−3.30 ± 2.90% (p = 1.000)) after 21 DIV.

Summarizing these observations, the elimination of the four ECM molecules TnC, TnR, Neurocan and brevican led to a reduction of inhibitory synapses on PNN-positive as well as PNN-negative knockout neurons cultivated for 14 DIV. This effect levelled off after three weeks where wildtype neurons maintained in the presence of knockout astrocytes displayed an enhanced number of vGAT-positive afferents and inhibitory synapses, irrespective of the presence or absence of PNNs (Fig. 2g,h).

### Higher neuronal network activity in the quadruple knockout mouse

**Figure 3:**
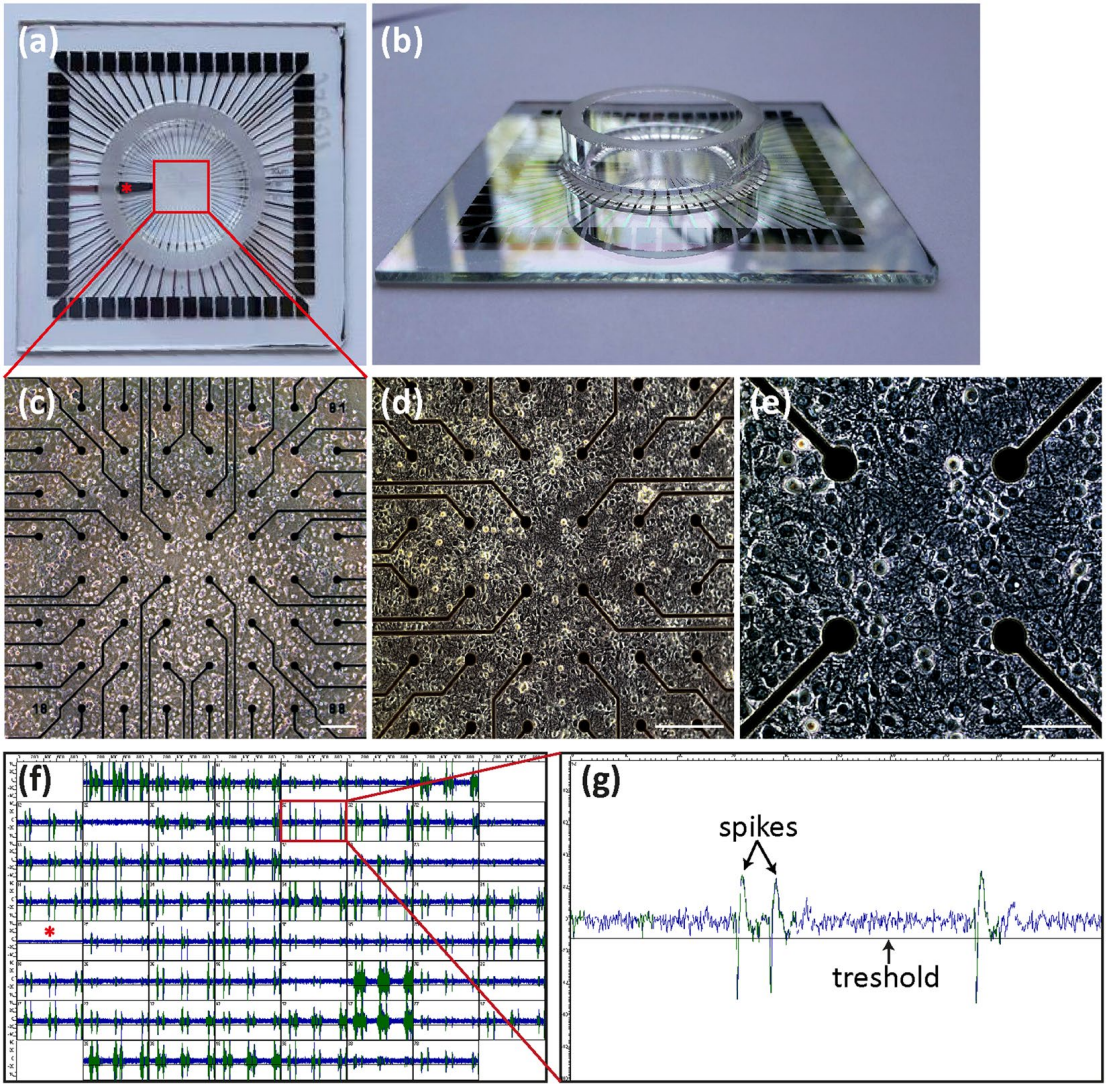
Cultivation of hippocampal neurons on MEAs. (**a**,**b**) Illustrates a standard MEA (electrode grid 8 × 8) for measuring the network activity of cells, e.g. of hippocampal neurons. In the centre of the MEA a glass ring (6 mm high) is located containing the electrode field on which the neurons were cultivated in 1 ml medium. Each MEA is equipped with 60 electrodes, including one internal reference electrode (red star in A). Each electrode is made of titanium nitride and has a diameter of 30 µm. The spacing between each electrode is 200 µm. **(c**,**d**,**e)** The exemplary microscopic images illustrate the growth of the hippocampal neurons on the MEA that elaborate a complex network after 14 DIV, as shown at higher magnification. **(e)** Neurons form numerous synaptic connections with each other. Scale bar **(c**,**d)**: 200 µm. Scale bar **(e)**: 100 µm. **(f)** Representative image of the activity of 60 electrodes shown by the spike sorter. Each small square displays the activity of one individual electrode. The occurrence of bursts can be detected through the emergence of simultaneous activity at the majority of electrodes. The red star marks the reference electrode. **(g)** Higher magnification of the neuronal activity of a singular electrode. Action potentials occur in the form of spikes and were registered when their amplitude exceeded the triggering threshold by 4.5-fold of the standard deviation.

To test the influence of the altered proportion of excitatory and inhibitory synapses at hippocampal quadruple knockout neurons on the spontaneous activity, MEA analysis was conducted as established in our laboratory^32^. Via the 60 electrodes of the MEA (Fig. 3a,b), the spontaneous activity of the neuronal network developing *in vitro* was analysed (Fig. 3c,d,e). Each electrode was individually recorded and the traces (Fig. 3g) were represented in small boxes (Fig. 3f,g), reflecting the spontaneous activity as well as the occurrence of bursts in different areas of the network in culture (Fig. 3c–e).

**Figure 4:**
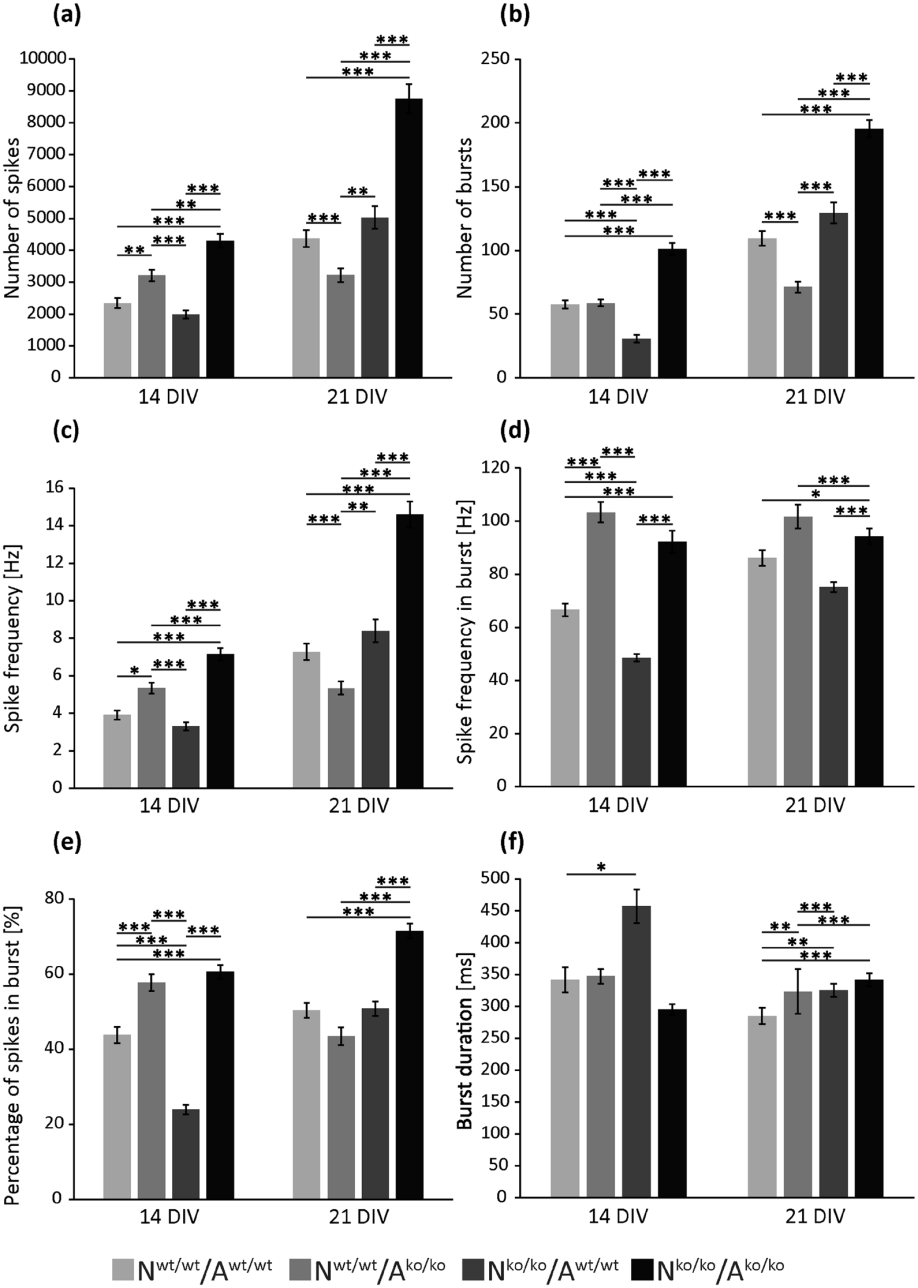
Spontaneous neuronal network activity after 14 and 21 DIV measured via MEA. Using MEA analysis, the spontaneous activity of neuronal networks derived from the hippocampus of either wildtype or quadruple knockout mice was examined. Different parameters were detected and quantified for the four conditions (N^wt/wt^/A^wt/wt^, N^wt/wt^/A^ko/ko^, N^ko/ko^/A^wt/wt^, N^ko/ko^/A^ko/ko^) including the number of spikes **(a)**, the number of bursts **(b)**, the spike frequency **(c)**, the spike frequency in bursts **(d)**, the percentage of spikes in bursts **(e)** and the mean burst duration **(f)**. Most analysed parameters were enhanced in the neuronal networks of knockout neurons cultured with knockout astrocytes (N^ko/ko^/A^ko/ko^) compared to the control (N^wt/wt^/A^wt/wt^). For example the number of spikes increased significantly to almost twice the control level after 14 DIV as well as 21 DIV and the number of bursts in culture was significantly enhanced too. This also resulted in a higher spike frequency as well as in a higher percentage of spikes in bursts in the knockout neurons grown with knockout astrocytes. The only parameter that remained unchanged between these two conditions was the mean burst duration. Statistics: Five independent experiments (N = 5) were performed with all in all 60 electrodes (n = 60) on each of one or two MEAs per condition. Data are expressed as mean ± SEM (Kruskal-Wallis test, p ≤ 0.05).

The number of spikes (Fig. 4a) which correspond to action potentials was significantly increased in knockout neurons co-cultured with knockout astrocytes (N^ko/ko^/A^ko/ko^) after 14 DIV (4306.84 ± 217.87 (p < 0.001)) and 21 DIV (8762.16 ± 454.98, p < 0.001) compared to the control (N^wt/wt^/A^wt/wt^) (14 DIV: 2348.39 ± 153.26, 21 DIV: 4367.73 ± 261.28). The same applied for the number of bursts (Fig. 4b) in this condition with a value of 101.04 ± 4.78 bursts per 10 min (p < 0.001) after 14 DIV and 195.45 ± 6.82 bursts (p < 0.001) after 21 DIV in comparison to the control values of 57.6 ± 3.22 bursts after 14 DIV and 109.38 ± 5.75 bursts after 21 DIV. Bursts consist of single spikes and occur simultaneously at a variety of electrodes in a short time interval. When knockout neurons were combined with wildtype astrocytes (N^ko/ko^/A^wt/wt^), the spike number was nearly the same as for the control, indicating a contribution of the astrocytic phenotype into the process of network activity. Similarly, the spike frequency was upregulated in cultures with knockout neurons co-cultivated with knockout astrocytes at both examined time points (14 DIV: 7.15 ± 0.33, p < 0.001; 21 DIV: 14.60 ± 0.70, p < 0.001) compared to the control (14 DIV: 3.91 ± 0.24; 21 DIV: 7.28 ± 0.43). With progressing cultivation time, the neuronal networks gained complexity, more bursts arose and a higher spike frequency in bursts emerged. Knockout neurons grown in the presence of knockout astrocytes (N^ko/ko^/A^ko/ko^) showed a significantly higher spike frequency in bursts of 92.18 ± 4.23 Hz (p < 0.001), which occurred after 14 DIV compared to 66.61 ± 2.44 Hz of the control (N^wt/wt^/A^wt/wt^). When cultivated for 21 DIV, these differences between the two conditions were still present (N^wt/wt^/A^wt/wt^: 86.17 ± 2.97, N^ko/ko^/A^ko/ko^: 94.32 ± 2.95 (p = 0.031). Interestingly, knockout neurons co-cultured with wildtype astrocytes (N^ko/ko^/A^wt/wt^) showed an opposite effect concerning their spike frequency in bursts, namely a reduction with a value of 48.54 ± 1.38 Hz (p < 0.001) after 14 DIV. After 21 DIV no significant difference could be observed (75.15 ± 1.91 Hz (p = 0.347)).

A higher amount of spikes in bursts appeared in the (N^ko/ko^/A^ko/ko^) condition compared to the control condition. Whereas in the control condition only 43.79 ± 2.18% spikes after 14 DIV and 50.37 ± 2.02% spikes after 21 DIV appeared within a burst, 60.62 ± 1,85% (p < 0.001) spikes in bursts after 14 DIV and 71.50 ± 1,90% (p < 0.001) spikes in bursts after 21 DIV occurred in the (N^ko/ko^/A^ko/ko^) condition. There was again a significant decrease of this analysed parameter in cultures of knockout neurons cultured with wildtype astrocytes (N^ko/ko^/A^wt/wt^) after 14 DIV with a percentage of 23.96 ± 1.31% (p < 0.001) spikes in burst, which attained control levels after 21 DIV, with a percentage of 50.78 ± 1.93% (p = 1.000). The duration of bursts was also measured using MEA analysis (Fig. 4f, Supplementary Table S3a,b). In the control condition (N^wt/wt^/A^wt/wt^) an average burst lasted for 341.88 ± 19.57 ms after 14 DIV and for 284.94 ± 12.83 ms after 21 DIV. No change in the mean duration of a burst was seen for knockout neurons grown with knockout astrocytes (N^ko/ko^/A^ko/ko^) after 14 DIV, which showed similar results of 294.96 ± 8.84 ms (p = 0.057) after 14 DIV and 341.72 ± 10.66 ms (p < 0.001) after 21 DIV, which also applies for all other conditions compared to the control condition. After 21 DIV, all conditions showed a significant rise in the burst duration in comparison to the control. The only striking difference of the burst duration was observable after 14 DIV for knockout neurons cultured with wildtype astrocytes (N^ko/ko^/A^wt/wt^). Here, the burst duration was significantly protracted to 456.95 ± 26.44 ms (p = 0.032) instead of 341.88 ± 19.57 ms as detected in the control.

In conclusion, the MEA analysis revealed that the deletion of four critical ECM constituents resulted in an increased network activity in the cultures of hippocampal neurons.

### Reduction of PNNs *in vivo* in the quadruple knockout mouse

**Figure 5:**
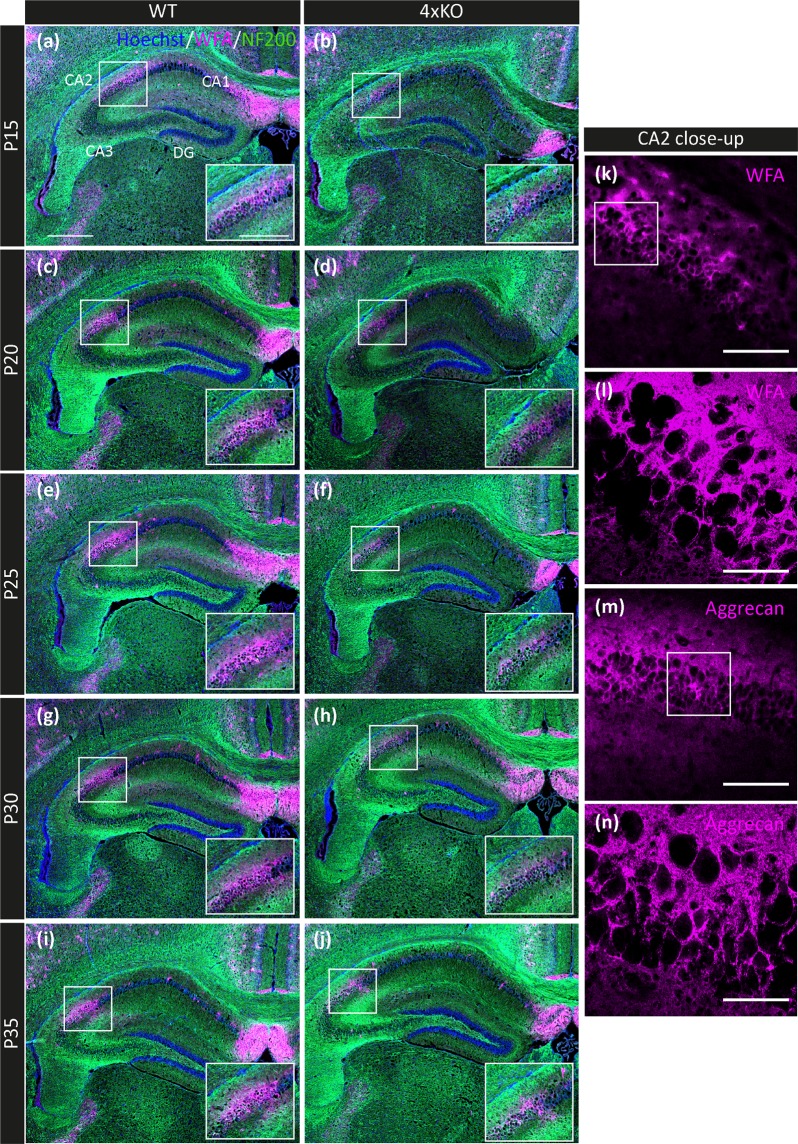
Expression of PNNs in the developing murine hippocampus *in vivo*. (**a**–**j**) Immunohistochemical detection of PNNs in the hippocampus of postnatal wildtype and knockout mice (P15, P20, P25, P30, P35) using the lectin WFA as marker for PNNs (magenta). Beyond a general expression of PNNs next to the CA1 region and single WFA-positive cells in the hippocampus as well as the cortex, a distinct PNN area was detected in the CA2 region of the hippocampus. This PNN area is noticeably smaller in the hippocampus of the quadruple knockout mice, which is also clearly visible in the higher magnification of this region within each image. The antibody NF200 (green) was utilized for the detection of neurons. The nuclei were marked by bisbenzimide (blue, Hoechst). Scale bar: 500 µm in a and 250 µm in inset of a. (**k**–**n**) Close-up images of the immunohistochemical WFA and aggrecan staining in the wildtype CA2 region of P20 mice. Images were taken using a confocal laser scanning microscope with a 400× (**k**,**m**) and 630x magnification (**l**,**n**). The both PNN markers aggrecan and WFA clearly show a strong fluorescence signal around the soma of CA2 neurons. Scale bar: 100 µm in k,m and 50 µm in l,n.

The ECM molecules TnC, TnR, brevican and neurocan are constituents of PNNs, therefore it appeared of interest to analyze the impact of the elimination of these genes on the formation of PNNs *in vivo*. The marker WFA was used to visualize PNNs in frontal brain slices of the hippocampus of wildtype (Fig. 5a,c,e,g,i) and knockout mice (Fig. 5b,d,f,h,j).

**Figure 6:**
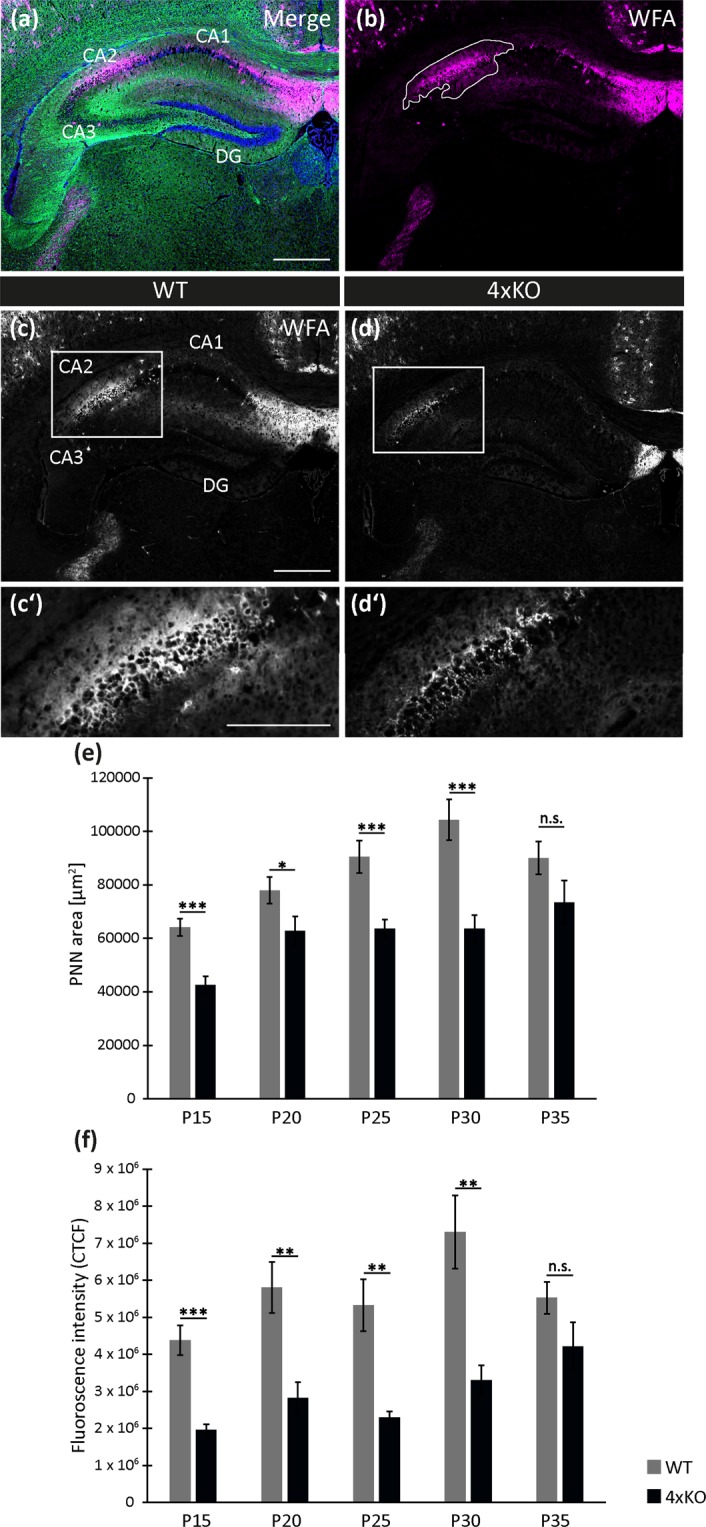
PNN area size in different developmental stages of the murine hippocampus. (**a**,**b**) Representative images illustrate the immunohistochemical detection of PNNs in the hippocampus of a P30 wildtype mouse. WFA (magenta) was used as a marker to detect the PNNs surrounding individual neurons. The area size in the CA2 region of the hippocampus was measured as indicated by the line surrounding the PNN-positive territory (b, white line). Neurons were visualized using an antibody to NF200 (green) and Bisbenzimide (Hoechst) was used to detect the nuclei. Scale bar in A: 500 µm. (**c**,**c’**, **d**,**d’**) Representative images of the WFA-staining converted into white for a better visualization of the PNN fluorescence intensity of the wildtype (**c**,**c’**) and knockout (**d**,**d’**) hippocampus at P25. The higher magnification of the WFA-positive area in the CA2 region (white box) indicates a higher intensity of the PNN fluorescence in the wildtype compared to the knockout hippocampus. Scale bar: 500 µm in C, D and 250 µm in C’, D’. (**e**) Quantitative *in vivo* analysis of the PNN area size in the hippocampal CA2 region of postnatal mice (P15, P20, P25, P30 and P35). Except at P35, all other analysed postnatal stage of the hippocampus showed a significantly decreased area size of the PNNs in the CA2 region of knockout mice. At P35, the PNN areas size was not significantly different between knockout and wildtype. (f) Quantitative analysis of the PNN fluorescence intensity (CTCF) in the CA2 region of the hippocampus of mice at P15, P20, P25, P30 and P35. The fluorescence intensity was significantly reduced within the hippocampus of knockout compared to wildtype mice. The only exception was at P35, when no significant alteration of the PNN fluorescence intensity was detectable. Statistics: Three independent experiments (biological replicates N = 3) were performed and the PNN area size of four hippocampi (n = 4) was examined. Data are expressed as mean ± SEM (F-test and unpaired student’s t-test, p ≤ 0.05).

The analysis of the PNNs *in vivo* was focused on the hippocampus of 15, 20, 25, 30 and 35 days old mice. Within the CA2 region of the hippocampus, an area consisting of numerous PNNs developed starting at P15 and evolved further along with the maturating hippocampus. This PNN territory was present in the wildtype as well as in the knockout hippocampus. A small proportion of the fluorescence in the CA2 region could be due to the diffuse interstitial matrix. However, it has recently been demonstrated that pyramidal neurons in the CA2 region are a major source of perineuronal nets in the hippocampus and positive for WFA and aggrecan^33^. This agrees with recent studies that emphasize that the WFA signal in CA2 is PNN-specific^37,38^. Consistent with this interpretation, a higher magnification image obtained using confocal laser scanning microscopy revealed that the major part of the fluorescence was concentrated in PNNs around neuronal cell bodies, suggesting a specific labeling of these structures (Fig. 5k–n). The PNN areas were determined for the different postnatal stages of the developing hippocampus (P15-P35) by measuring the WFA-positive area (Fig. 6). The quantification revealed a significant downregulation of the PNN area in the quadruple knockout hippocampus at P15, P20, P25, and P30 (Fig. 6e). The PNN area amounted to 42558.46 ± 3223.24 µm^2^ (p < 0.001) at P15, 62810.40 ± 5409.42 µm^2^ (p = 0.05) at P20, 63750.31 ± 3300.47 µm^2^ (p < 0.001) at P25 and 63620.73 ± 5082.00 µm^2^ (p < 0.001) at P30 compared with the values of the wildtype of 64155.52 ± 3273.71 µm^2^ at P15, 78005.23 ± 5021.09 µm^2^ at P20, 90590.67 ± 6055.96 µm^2^ at P25 and 104404.09 ± 7588.34 µm^2^ at P30 (Supplementary Table S4a,b). In the wildtype hippocampus, the area size increased over time until P30. No significant difference of the PNN size was detectable between the wildtype and knockout hippocampus at P35. At this stage the PNN area of the knockout hippocampus amounted to 73468.03 ± 8223.63 µm^2^ and of the area size of the wildtype hippocampus encompassed 90108.04 ± 6145.99 µm^2^ (p = 0.120), indicating an alignment of their PNN area size towards adulthood.

Next to the area size, the corrected total cell fluorescence (CTCF) of the WFA-positive area in the CA2 region of the hippocampus was measured (Fig. 6c,c’,d,d’,f). As previously seen for the area size, the fluorescence intensity was also significantly diminished in the quadruple knockout hippocampus at P15, P20, P25 and P30 (P15: 1963962.39 ± 150734.82, p < 0.001; P20: 2820496.51 ± 436309.19, p = 0.0018; P25: 2299008.92 ± 160585.48, p = 0.0012; and P30: 3305522.46 ± 395786.81, p = 0.002) compared to the wildtype situation (P15: 4380059.02 ± 403847.23, P20: 5805772.77 ± 691451.33, P25: 5328356.19 ± 702004.79 and P30: 7300182.00 ± 987441.78). At P35, no change with regard to the fluorescence intensity of the PNN area was found in the knockout hippocampus (4217469.63 ± 647443.36 in comparison to the wildtype hippocampus (5529794.88 ± 430019.63, p = 0.108). In conclusion, the maturation of PNNs is significantly delayed in the quadruple knockout hippocampus but normalized after postnatal day 30 to attain the wildtype control levels in adulthood (Supplementary Table S4a,b).

### Alterations in the gene expression in the quadruple knockout hippocampus at postnatal day 21

Considering the fact that the PNN maturation was significantly retarded in the P21 hippocampus of the quadruple knockout mouse, this time point was chosen for the comparative analysis of gene expression using microarrays.

**Figure 7:**
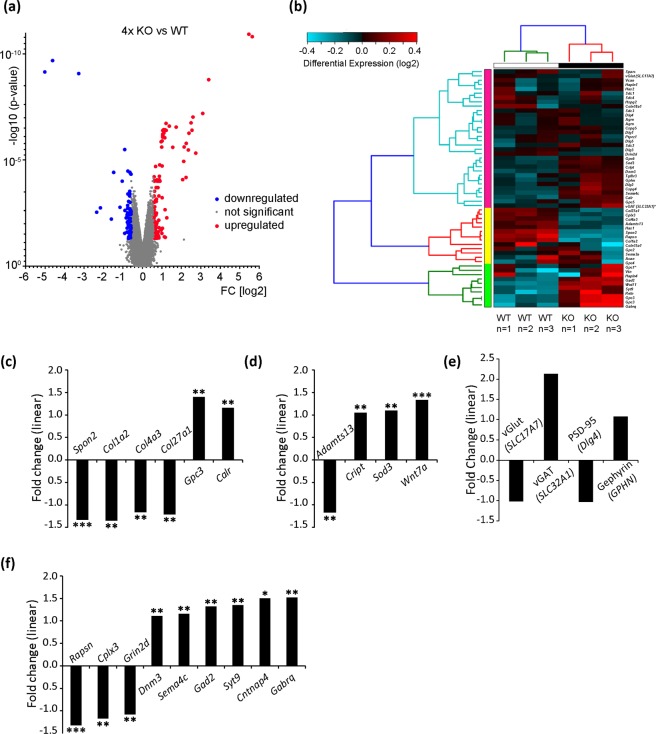
Hierarchical clustering of genes in the P21 hippocampus of quadruple knockout and wildtype mice. **(a)** The microarray analysis revealed that 438 genes are significantly altered in the quadruple knockout mouse. These are depicted in form of a volcano plot, which includes upregulated genes (red dots), downregulated genes (blue dots) and unaffected genes (grey). **(b)** The hierarchical clustering of genes in the P21 hippocampus uncovered a differential gene expression pattern in the quadruple knockout compared to the wildtype mouse using three dependent hippocampus samples of siblings (n = 3). The cluster heat map comprises significantly up- and downregulated genes of interest concerning the annotations “ECM” as well as “neurons/synapses” including *Gpc3*, *Gabrq*, *Gad2*, *Wnt7*, *Syt9*, *Sod3*, *Dnm3*, *Cript*, *Sema4c*, *Calr*, *Cplx3*, *Col4a3*, *Col27a1*, *Adamts13*, *Grin2d*, *Col1a2*, *Spon2* and *Rapsn*. The colour shift of light blue to light red indicates the expression change of these genes, that is genes that appear in blue were down- whereas genes illustrated in red were upregulated in the hippocampus of the quadruple knockout mouse. **(c)** Linear fold change of exemplary ECM molecules (*Spon2*, *Col1a2*, *Col4a3*, *Col27a1*, *Gpc3* and *Calr*) and **(d)** neuron related molecules (*Adamts13*, *Cript*, *Sod3*, *Wnt7a*) in the P21 quadruple knockout hippocampus. **(e)** Linear fold change of representative genes belonging to the field of neurons and their synapses (*Rapsn*, *Cplx3*, *Grin2d*, *Dnm3*, *Sema4c*, *Syt9*, *Gad2*, *Cntnap4*, *Gabrq*, *SLC17A7*, *SLC32A1 and GPHN*) in the quadruple knockout hippocampus.

Overall 34473 genes were examined of which 438 were significantly altered in the quadruple knockout hippocampus. Because we had detected differences in synapse formation, we were particularly interested in genes associated with the ECM or synaptogenesis. A volcano plot and a clustering heatmap highlight the dysregulated genes of interest of these functional annotations, which are either up- or downregulated (Fig. 7a,b) in the knockout hippocampus. The significantly altered genes comprise *Gpc3*, *Gabrq*, *Gad2*, *Wnt7*, *Syt9*, *Sod3*, *Dnm3*, *Cript*, *Sema4c*, *Calr*, *Cplx3*, *Col4a3*, *Col27a1*, *Adamts13*, *Grin2d*, *Col1a2*, *Spon2* and *Rapsn*. For example, the three collagens *Col1a2* (−1.35-fold, p = 0.0017), *Col4a3* (−1.16-fold, p = 0.0014) and *Col27a1* (−1.21-fold, p = 0.0098) were significantly downregulated, as well as *Spon2* (−1.33-fold, p = 0.0002) (Fig. 7c). *Spon2* is known to facilitate neurite outgrowth of hippocampal neurons^39,40^. In contrast, the ECM genes *Gpc3* (1.40-fold, p = 0.0089) and *Calr* (1.16-fold, p = 0.0038) were significantly upregulated in the quadruple knockout hippocampus. Interestingly, genes related to neuronal development and function (Fig. 7d) were also modified, as shown by a decreased linear fold change of *Adamts13* (−1.17-fold, p = 0.0058) and increased values for the linear fold change of *Cript* (1.05-fold, p = 0.0083), *Sod3* (1.10-fold, p = 0.0081) and *Wnt7a* (1.33-fold, p = 0.0002). The expression of the neuronal genes (Fig. 7f) *Cplx3* (−1.17-fold, p = 0.0037) and *Grin2d* (−1.08-fold, p = 0.0057) was significantly diminished, whereas *Dnm3* (1.11-fold, p = 0.0015), *Sema4c* (1.16-fold, p = 0.0065), *Gad2* (1.32-fold, p = 0.0062), *Cntnap4* (1.50-fold, p = 0.0198) and *Gabrq* (1.52-fold, p = 0.0310) were significantly enhanced. However, the expression of mRNA for the synaptic components *SLC17A7* (encoding for vGlut: −1.02-fold, p = 0.994), *SLC32A1* (encoding for vGAT: 2.13-fold, p = 0.778), *Dlg4* (encoding for PSD-95: −1.03, p = 0.898) and *Gphn* (encoding for Gephyrin: 1.08, p = 0.049) was not significantly altered (Fig. 7e). Chondroitin sulfate proteoglycans such as *Cspg4*, *Cspg5* or *Ptprz1* that could potentially compensate the knockout were also analysed, but no significant difference could be observed in their expression levels (Fig. 7b). A tabular overview of the data can be found in the supplement (Supplementary Table S5).

## Discussion

Although it is known for a few years that PNNs contribute to the process of synaptogenesis, it is not yet clear to which extent the different molecules are involved therein^9,10,41^.

Because of the manifold and complex character of the PNN structure, it is not surprising that neurons with PNN, as well as quadruple knockout neurons with a defective PNN composition display considerable changes in their synapse formation. Several studies of the recent years have shown that one of the main features of the ECM and PNNs is to restrict the formation of neurites^42^ and their synapses with concurrent protection of neurons towards harmful substances or events^43^. These studies relied on the application of the bacterial derived enzyme chondroitinase ABC (ChABC), which degrades the chondroitin-4-sulfate, chondroitin-6-sulfate, dermatan sulfate and the hyaluronan glycosaminoglycan chains^10,44^. When hippocampal neurons were treated with ChABC *in vitro*, synapse formation was boosted^6^. Different from the ChABC-treated cultures, the quadruple knockout neurons miss the CSPGs neurocan and brevican, but still express aggrecan, the PNN marker used in this study, and possibly other CSPGs such as phosphacan^45^. These may be expressed similar to the wildtype control because the gene array analysis did not reveal substantial modifications of CSPG genes.

TnR is a pivotal constituent of PNNs and its elimination leads to a change of PNN distribution, composition and function^46^. A recent study concerning hippocampal neurons from TnR-deficient mice reported a modified PNN organization around dendrites that could be rescued by adding TnR and aggrecan into the culture medium^21^. With regard to physiological parameters, TnR-knockout mice displayed an enhancement of excitatory synaptic transmission in the hippocampal CA1 region^47^. This is in agreement with our observation that an increased number of excitatory synapses resulted in enhanced synaptic transmission in the neuronal network of the quadruple knockout mouse, which was recorded via MEA analysis *in vitro*.

It is conceivable that the missing expression of TnC and TnR is compensated by other glycoproteins. For example, the tenascin family comprises the four genes TnC, TnR, TnW and TnX, but neither TnW nor TnX are elevated in the quadruple knockout mouse^48,49^. In contrast, the two glycoproteins fibulin-1 and -2 of the ECM are clearly upregulated on the protein level in the quadruple knockout brains. This is accompanied by an altered hyaluronan deposition in the mutant ECM^28^. Interestingly, fibulin-1 mutations are associated with cognitive deficits in the human and fibulin-2 mediates TGF-beta1 effects on adult neural stem cells^50,51^.

A former study already reported the synaptogenesis of hippocampal quadruple knockout neurons in an *in vitro* co-culture system. The loss of TnC, TnR, brevican and neurocan resulted in a significant increase of synaptic density within 14 DIV that transited to a decremented synapse number after 21 DIV. Neurons coated with PNNs were characterized by an attenuated synapse number after both 14 and 21 DIV^29^.

Unexpectedly, in our approach WT astrocytes were not capable to rescues the PNN formation by knockout neurons. Astrocytes *in vitro* secrete brevican, neurocan and TnC. However, TnC is not a major component of perineuronal nets^52^ while TnR is mainly expressed by oligodendrocytes and not by astrocytes^10^ (and is only present at low concentration in the co-culture of WT astrocytes with KO neurons, if at all. Lecticans do contain binding sites for hyaluronic acid and TnR^53^. Several reports suggest that CSPGs like aggrecan or brevican are incorporated into perineuronal nets through their interaction with TnR and hyaluronic acid^14,53,54^. However, TnR is absent from the knockout neurons in co-culture. This may explain why in our model the incorporation of astrocytic CSPGs into PNNs was impaired. This agrees with a study that emphasized the functional importance of TnR for the formation of PNNs^21^.

For the formation of structurally and functionally intact synapses, specific molecules are necessary. In former studies, it was demonstrated that the astrocyte-secreted factors thrombospondin and hevin lead to the development of structural, but silent synapses^55,56^. In line with this, Gpc4 and Gpc6 were recently also discovered as astrocytic factors, which stimulate the excitatory synapse formation by regulating glutamate receptor clustering^57^. The analysis of the gene expression performed in this study revealed an upregulated expression of Gpc3 in the hippocampus of P21 quadruple knockout mice, which is probably an explanation for the higher number of excitatory synapse number and the elevated activity in the quadruple knockout cultures.

A recently published study has demonstrated an altered synaptic plasticity in the dorsal dentate gyrus of the quadruple knockout hippocampus that translated into an enhanced short-term depression in conjunction with a modified frequency-dependence *in vivo*^30^. This changed synaptic plasticity *in vivo* is also detected in *in vitro* neuronal networks performed in this study. Here, the spontaneous network activity was significantly increased in quadruple knockout cultures. These data are in agreement with the report of Dityatev and colleagues who found a higher excitability of hippocampal neurons in culture after ChABC treatment^14^. Furthermore, hippocampal neuronal cultures treated with hyaluronidase developed an epileptiform activity due to the occurrence of superbursts^58^. Thus, it is evident that the loss of pivotal ECM molecules leads to a higher neuronal activity independent of whether the ECM molecules are digested by appropriate enzymes or genetically eliminated.

The initiation of PNN formation in the murine hippocampus detectable by WFA occurred between postnatal stage P10 and P15. In the adult hippocampus, the PNN formation resembled the expression in the wildtype hippocampus, indicating a progressive compensation of the four missing ECM molecules TnC, TnR, brevican and neurocan, possibly by the upregulation of other ECM molecules. The results found for the *in vivo* PNN formation fit perfectly with the *in vitro* findings^29^, both demonstrating a diminished PNN formation in the postnatal quadruple knockout mouse. A recent study demonstrated that especially the CA2 excitatory pyramidal neurons are surrounded by PNNs. When mice were kept in an enriched environment, the PNN expression was enhanced, indicating a correlation between PNN development and environmental stimuli within the juvenile brain^33^. Interestingly, PNNs have been proposed as indispensable mediators of the modulation of synaptic plasticity by the transcription factor Otx2^59–61^. An impaired PNN formation and structure accompanied with altered synaptic plasticity is associated with a couple of psychiatric and neurological diseases such as schizophrenia and epilepsy, as reviewed recently^62^.

Significant dysregulation of genes of interest of the ECM or genes associated with neuron biology were detected by a comparative transcriptome analysis of the P21 quadruple knockout compared to the wildtype hippocampus using microarrays.

Like the previously discussed Gpc3 also other molecules contribute to synapse formation and synaptic transmission. For example, in the quadruple knockout mouse *Sema4c* is upregulated, which is known as interaction partner of different PSD95 isoforms in neocortical cultures as well as in the adult brain of mice^63^. Cplx3 that was significantly downregulated in the quadruple knockout hippocampus regulates the synaptic neurotransmitter release of both excitatory and inhibitory synapses^64^. The molecule Grin2d, also known as GluN2D, was reduced in the quadruple knockout hippocampus. By using Grin2d-deficient mice, its contribution in synaptic transmission of hippocampal interneurons of young mice as well as of the pyramidal CA1 neurons of newborn pups has been proven^65^. Furthermore, Grin2d has been found in hippocampal interneurons and enhances their activity due to its involvement in synaptic transmission^66^.

Thus, available data concur with our observation that a modified number of excitatory and inhibitory synapses prevail in quadruple knockout neurons *in vitro*. The excitation versus inhibition balance is considered very important in the context of neuropsychiatric disease^67^. A shift of the balance can result from neurodevelopmental abnormalities targeting the inhibitory interneurons^68^ or from structural changes at the synapse^69^. Our observations suggest that the ECM intervenes in the balance of excitation and inhibition in neural networks. A recent review elegantly summarizes potential mechanisms of action of PNNs^70^. Brevican for example, which is missing in our mouse model controls the location of AMPA and potassium channels and might therefore affect synapse dynamics^71^. Neurocan is known to influence the binding of neural cell adhesion molecule (NCAM) and ephrin type-A receptor 3 (EPHA3) at perisomatic synapses^72^. TnR, another protein missing in the quadruple knockout mouse is known to be associated with HNK-1 and necessary for the maintenance of the excitatory and inhibitory balance in CA1 region^47^. Beyond these PNN-associated compounds also proteins released by astrocytes influence synaptogenesis, for example thrombospondins and the gabapentin receptor^55,73^, Hevin and SPARC^56^, or Pentraxin 3 that promotes excitatory synapse formation by fostering the clustering of AMPA glutamate receptors^74^. In the light of these observations, the interactions of ECM molecules with neuronal receptors may represent a promising theme for analyzing this issue in future studies.

## Methods

### Animal housing and ethical standards

The present study was carried out in accordance with the European Council Directive of September 22, 2010 (2010/63/EU) for care of laboratory animals and approved by the animal care committee of North Rhine-Westphalia, Germany, based at the LANUV (Landesamt für Umweltschutz, Naturschutz und Verbraucherschutz, Nordrhein-Westphalen, D-45659 Recklinghausen, Germany). The study was supervised by the animal welfare commissioner of Ruhr-University. Male and female SV129 mice were housed individually with a constant 12-h light-dark cycle and access to food and water *ad libitum*. All efforts were made to reduce the number of animals in the experiments. Embryos of both sexes were used.

### Cell culture

#### Co-cultivation of astrocytes and neurons

Hippocampal neurons were obtained from embryonic mice and co-cultured with astrocytes in an indirect contact as described previously^31^ (See supplemental data S6).

### Multielectrode array (MEA)

#### Cultivation of hippocampal neurons on MEAs

Electrophysiological recordings of neuronal networks were performed using multielectrode arrays (MEAs) (60MEA200/30iR-Ti, Multi Channel Systems MCS GmbH) following the previously described protocol^32,75^.

#### Electrophysiological recordings

Recordings of the spontaneous activity were performed for 10 min with a sampling frequency of 20 kHz. The data of the individual electrodes were collected using the software MC_Rack (version 3.9.0 by Multi Channel Systems MCS GmbH). The field potentials of raw data were corrected with a high pass filter which had a frequency of 200 Hz. The spontaneous activity was detected with a spike detector only if the value exceeded a threshold value 4.5-fold higher than the standard deviation. The following parameters were set for the measurements: Maximal interval to start burst, 10 ms; Maximal interval to end burst, 100 ms; minimal interval between bursts, 210 ms; minimal duration of burst, 50 ms; minimal number of spikes in bursts, 5.

### Immunocytochemistry

#### Staining of excitatory synapses

Initially, the neurons were fixed with 4% (w/v) PFA (Carl Roth GmbH & Co. KG; Cat. No.: 4235.1) for 10 min at RT. After fixation the cells were washed four times for 5 min each with wash solution (PBS, 10% v/v fetal bovine serum (FBS (Sigma-Aldrich by Merck KGaA; Cat. No.: F7524), 0.1 mM glycine (VWR International GmbH; Cat. No.: 101196X), 0.1% v/v Triton X-100 (AppliChem GmbH; Cat. No.: A4975,0500)). The primary antibodies were diluted in wash solution as follows: vGlut1, polyclonal, guinea pig, 1:500 (Synaptic Systems GmbH; Cat. No.: 135304, RRID: AB_887878); PSD95, IgG_2a_, mouse, 1:500 (Merck KGaA; Cat. No.: MAB1598, RRID: AB_1121285) and aggrecan, polyclonal, rabbit, 1:500 (Merck KGaA; Cat. No.: AB1031, RRID: 90460). The incubation was carried out for 1 h at room temperature. Afterwards, the secondary antibodies were diluted in wash solution and were incubated for 1 h at RT: anti-guinea pig, IgG (H + L), AF647, 1:500 (Dianova; Cat. No.: 106-605-003, RRID: AB_2337446), anti-mouse, IgM + IgG (H + L), AF488, 1:250 (Dianova; Cat. No.: 115-545-044, RRID: AB_2338844) and anti-rabbit, IgG (H + L), Cy3, 1:500 (Dianova; Cat. No.: 111-165-045, RRID: AB_2338003).

#### Staining of inhibitory synapses

The staining procedure of inhibitory synapses was similar to the previously described protocol by Dobie and colleagues^36^. Neurons were fixed after 14 and 21 days *in vitro* with 4% (w/v) PFA for 10 min at RT. The primary antibodies were diluted in 3% w/v BSA (in PBS) (vGAT, polyclonal, guinea pig, 1:1000 (Synaptic Systems GmbH; Cat. No.: 131 004, RRID: AB_887873), gephyrin, IgG_1_, mouse, 1:500 (Synaptic Systems GmbH; Cat. No.: 147 011, RRID: AB_887717) and aggrecan, polyclonal, rabbit 1:500 (Merck KGaA; Cat. No.: AB1031, RRID: 90460). The secondary antibodies were diluted in 3% w/v BSA (in PBS), as follows: anti-guinea pig, IgG (H + L), AF647 (Dianova; Cat. No.: 106-605-003, RRID: AB_2337446), 1:500; anti-mouse, IgM + IgG (H + L), AF488 (Dianova; Cat. No.: 115-545-044, RRID: AB_2338844), 1:250 and anti-rabbit, IgG (H + L), Cy3, 1:500 (Dianova; Cat. No.: 111-165-045, RRID: AB_2338003).

### Immunohistochemistry

#### Preparation of cryosections

For the preparation of cryosections, wildtype and quadruple knockout mice were intracardially perfused according to the German animal protection laws and approved by the responsible governmental authorities (Landesamt für Umweltschutz, Naturschutz und Verbraucherschutz (LANUV), Nordrhein-Westfalen, D-45659 Recklinghausen, (AZ 84-02.04.2015.A014)).

With the help of a peristaltic pump (Cole-Parmer GmbH), the blood was exchanged with a 0.9% (w/v) NaCl solution (Carl Roth GmbH & Co. KG) with 0.2% (v/v) of 25 000 I.E./5 ml heparin-sodium (Ratiopharm GmbH). Thereafter, 4% w/v PFA was applied for 10–15 min for fixation of the brain. Decapitation as well as the quick removal of the brain from the skull followed. The brain was transferred into 4% w/v PFA and incubated overnight at 4 °C. On the next day, the brain was placed into 30% w/v sucrose (Thermo Fisher Scientific Inc.). After approximately two days, the brain was embedded with tissue tec freezing medium (Leica Microsystems GmbH; Cat. No.: 14020108926) on dry ice and stored at −20 °C. Afterwards, the frozen brains were cut into 14 µm thick frontal sections (interaural: 1.86 mm, bregma: −1.94 mm) using a cryostat CM 3050 S (Leica Microsystems GmbH).

#### Staining of cryosections

First, the cryosections were incubated in blocking solution for 1 h at RT in a wet chamber, followed by the incubation with primary antibodies. The primary antibodies were diluted in PBT1 (PBS, 0.1% w/v BSA, 0.1% v/v Triton X-100) containing 5% v/v goat serum (Dianova) as follows: neurofilament 200 (NF200), polyclonal, rabbit, 1:300 (Sigma by Merck KGaA; Cat. No.: N4142, RRID: AB_477272), *Wisteria floribunda* agglutinin (WFA, 1:100 (Sigma by Merck KGaA; Cat. No.: L1516)) and aggrecan, polyclonal, rabbit, 1:300 (Merck KGaA; Cat. No.: AB1031, RRID: 90460). An overnight incubation at 4 °C was carried out and on the next day, sections were washed three times with PBS. Afterwards, the secondary antibodies as well as Hoechst dye (1:100000) were diluted in PBS/A (PBS, 0.1% w/v BSA) as follows: anti-rabbit, IgG (H + L), AF488, 1:250 (Dianova; Cat. No.: 111-545-045, RRID: AB_2338049), anti-streptavidin, Cy3, 1:500 (Dianova; Cat. No.: 016-160-084, RRID: AB_2337244) and anti-rabbit, IgG (H + L), Cy3, 1:500 (Dianova; Cat. No.: 111-165-045, RRID: AB_2338003). Sections were incubated with the secondary antibodies for 3 h at RT in a wet darkened chamber. In a last step, the cryosections were washed three times with PBS and once with MilliQ water and finally mounted with coverslips (24 × 50 mm, Menzel by Thermo Fisher Scientific Inc.) using ImmuMount.

### Molecular biology

#### mRNA isolation

The GenElute Mammalian Total RNA Miniprep Kit (Sigma-Aldrich by Merck KGaA; Cat. No.: RTN350) was used to isolate the mRNA from the hippocampal tissue following the manufacturer’s instructions.

#### Affymetrix GeneChip® analysis

Using Affymetrix GeneChip® Mouse Gene 2.0 ST Arrays, a whole-transcript expression analysis of >30,000 RefSeq transcripts of P21 hippocampus of wildtype and quadruple knockout mice siblings was performed in triplicates. RNA quantity was assessed using the NanoDrop 1000 (Thermo Scientific Nano Drop Technologies), and RNA quality control was carried out with the RNA 6000 Nano Assay (Agilent 2100 Bioanalyser) to ensure that the samples had an RNA integrity number (RIN) of at least 9. All further processing of total RNA was performed according to the Ambion whole-transcript Expression kit and the Affymetrix GeneChip whole-transcript terminal labeling and control kit manuals. The cDNA fragment size was checked using the 2100 Bioanalyser (fragment size between 50 and 200 bp). Fragmented sense cDNA was biotin-endlabelled with terminal deoxynucleotidyl transferase and samples were hybridised to GeneChip® Mouse Gene 2.0 ST Arrays at 45 °C for 16 hours with 60 rpms. Hybridised arrays were then washed and stained on a Fluidics Station 450 (program: FS450 0002) and scanned on a GeneChip® Scanner 3000 7 G (both Affymetrix). Raw image data were analysed with GeneChip^®^ Command Console^®^ Software (AGCC). Gene expression intensities were normalized (Robust Multichip Average^76^) and summarized using Affymetrix® Expression Console™ Software. Transcripts that were differently expressed more than 1.5-fold with an Anova p-value less than 0.05 between the analysed samples were addressed as regulated.

The heat-Map was established using AltAnalyse^77^ version 2.1.0 with following parameters: cosine hopach for column clustering and cosine weighted for row clustering. The rows were normalized relative to the row median. The volcano plot was constructed using Graph Pad Prism 6.

### Experimental design and statistical analysis

In general, the data are expressed as mean ± SEM. The statistical significance is given by the p value: p ≤ 0.05 = *, p ≤ 0.01 = ** and p ≤ 0.001 = ***. The program IBM SPSS Statistic (Version 20) was used for statistical evaluation. The exact number of experimental repetitions can be found in the figure legends.

### Microscopy

The confocal laser-scanning microscope LSM 510 meta operated with the ZEN2009.Ink software (Carl Zeiss Microscopy GmbH) was used for image acquisition of the immunocytochemical stainings. Different layers were recorded with an interval of 0.25 µm using 630-fold magnification. For generating two-dimensional images, pictures were overlaid. For quantification of synaptic puncta, the program ImageJ was used with the Plugin “Puncta Analyser” from Barry Wark; licensed under http://www.gnu.org/copyleft/gpl.html) and the following settings were adjusted: rolling ball radius = 50 pixel, size (pixel^2^) = 2-infinity and circularity = 0.00–1.00.

#### PNNs *in vivo*

The immunological staining of the cryosections was recorded using the microscope Axio Zoom.V16 (Carl Zeiss Microscopy GmbH). A prominent PNN-positive area was found in the CA2 region of the hippocampus^33^. To quantify the WFA-intensity and -area, the PNNs in the micrographs were manually bordered, the corrected total cell fluorescence (CTCF) was determined as previously described^78^ and the area was measured using ImageJ.

## Supplementary information

Supplementary information

## Data Availability

All data generated or analysed during this study are included in this published article (and its Supplementary Information files). The microarray data was deposited in the gene Expression Omnibus (GEO accession: GSE127216).
