# Supplementary material for: Elimination of the four extracellular matrix molecules tenascin-C, tenascin-R, brevican and neurocan alters the ratio of excitatory and inhibitory synapses
Source: Sci Rep. 2019 Sep 26;9:13939. doi: 10.1038/s41598-019-50404-9 (PMC6763627; doi:10.1038/s41598-019-50404-9)
Supplement: Supplementary file 1 — Supplementary information [file 41598_2019_50404_MOESM1_ESM.pdf]

Elimination of the four extracellular matrix molecules tenascin-C, tenascin-R, brevican and neurocan alters the ratio of excitatory and inhibitory synapses

Authors:

Christine Gottschling[1], David Wegrzyn[1], Bernd Denecke[2], Andreas Faissner[1]

1) Department of Cell Morphology and Molecular Neurobiology,

2) IZKF Aachen, RWTH Aachen, D-52074 Aachen, Germany

Supplementary Information

Supplementary Table S1a: The average number of excitatory synaptic puncta ± SEM and their percentage increase/decrease ± SEM compared to the $N^{wt/wt}/A^{wt/wt}$ – control for neurons with and without PNNs after 14 and 21 DIV

Condition	Genotype of astrocytes and neurons	Number of synaptic puncta ± SEM		
		vGlut1	PSD95	colocalization
14 DIV with PNNs	$N^{wt/wt}/A^{wt/wt}$	1657.6 ± 94.4	3007.1 ± 150.7	1195.9 ± 85.1
		0.0 ± 5.7 %	0.0 ± 5.0 %	0.0 ± 7.1 %
	$N^{wt/wt}/A^{ko/ko}$	1598.6 ± 87.9	2958.5 ± 191.7	1147.6 ± 80.5
		-3.6 ± 5.3 %	-1.6 ± 6.4 %	-4.1 ± 6.7 %
	$N^{ko/ko}/A^{wt/wt}$	1879.4 ± 108.6	3607.8 ± 194.6	**1761.3 ± 149.4**
		13.4 ± 6.6 %	20.0 ± 6.5 %	**47.3 ± 12.5 %**
	$N^{ko/ko}/A^{ko/ko}$	1937.7 ± 112.3	**3914.6 ± 174.0**	**1654.0 ± 121.9**
		16.9 ± 6.8 %	**30.2 ± 5.8 %**	**38.3 ± 10.2 %**
14 DIV without PNNs	$N^{wt/wt}/A^{wt/wt}$	1819.9 ± 103.6	3101.5 ± 120.7	1210.3 ± 73.5
		0.0 ± 5.7 %	0.0 ± 3.9 %	0.0 ± 6.1 %
	$N^{wt/wt}/A^{ko/ko}$	1964.4 ± 85.6	3265.2 ± 152.5	1238.4 ± 56.0
		7.9 ± 4.7 %	5.3 ± 4.9 %	2.3 ± 4.6 %
	$N^{ko/ko}/A^{wt/wt}$	**2304.8 ± 102.8**	**3881.1 ± 154.6**	**1812.2 ± 97.0**
		**26.7 ± 5,7 %**	**25.1 ± 5.0 %**	**49.7 ± 8.0 %**
	$N^{ko/ko}/A^{ko/ko}$	**2433.1 ± 124.1**	**4268.9 ± 156.5**	**1842.6 ± 103.0**
		**33.7 ± 6.8 %**	**37.6 ± 5.1 %**	**52.2 ± 8.5 %**
21 DIV with PNNs	$N^{wt/wt}/A^{wt/wt}$	2220.7 ± 116.5	3496.9 ± 171.3	1945.8 ± 122.5
		0.0 ± 5.3 %	0.0 ± 4.9 %	0.0 ± 6.3 %
	$N^{wt/wt}/A^{ko/ko}$	2157.3 ± 129.5	3692.2 ± 200.2	1993.0 ± 144.1
		-2.9 ± 5.8 %	5.6 ± 5.7 %	2.4 ± 7.4 %
	$N^{ko/ko}/A^{wt/wt}$	2532.4 ± 122.2	**4495.6 ± 186.8**	**2557.3 ± 153.9**
		14.0 ± 5.5 %	**28.6 ± 5.3 %**	**31.4 ± 7.9 %**
	$N^{ko/ko}/A^{ko/ko}$	**2782.3 ± 127.9**	**4349.3 ± 144.9**	**2549.5 ± 116.3**
		**25.3 ± 5.8 %**	**24.4 ± 4.1 %**	**31.0 ± 6.0 %**
21 DIV without PNNs	$N^{wt/wt}/A^{wt/wt}$	2629.9 ± 108.8	4254.4 ± 156.1	2165.2 ± 95.7
		0.0 ± 4.1 %	0.0 ± 3.7 %	0.0 ± 4.4 %

$N^{wt/wt}/A^{ko/ko}$	2568.9 ± 114.9	4025.1 ± 149.7	2082.9 ± 101.3
	-2.3 ± 4.4 %	-5.4 ± 3.5 %	-3.8 ± 4.7 %
$N^{ko/ko}/A^{wt/wt}$	2796.6 ± 123.6	4585.9 ± 162.7	2341.5 ± 108.0
	6.3 ± 4.7 %	7.8 ± 3.8 %	8.1 ± 5.0 %
$N^{ko/ko}/A^{ko/ko}$	2457.6 ± 84.9	3810.8 ± 134.6	1974.5 ± 78.2
	-6.6 ± 3.2 %	-10.4 ± 3.2 %	-8.8 ± 3.6 %

Synaptic puncta were visualized using laser scanning microscopy, the average number per neuron is given. The % increase/decrease of individual puncta in comparison to the control condition ($N^{wt/wt}/A^{wt/wt}$) was calculated with reference to the average number of puncta determined for the control. Significant differences are highlighted in bold character, the p-values are listed in Supplementary Table S1b .

Supplementary Table S1b: Table lists the p-values related to the altered numbers of excitatory synaptic puncta between the four conditions for neurons with and without PNNs after 14 and 21 DIV.

Condition	Genotypes	p-value vGlut1	p-value PSD95	p-value colocalization
14 DIV with PNNs	$N^{wt/wt}/A^{wt/wt}$ vs $N^{wt/wt}/A^{ko/ko}$	p=0.472	p=1.000	p=1.000
	$N^{wt/wt}/A^{wt/wt}$ vs $N^{ko/ko}/A^{wt/wt}$	p=0.185	p=0.392	p=0.050
	$N^{wt/wt}/A^{wt/wt}$ vs $N^{ko/ko}/A^{ko/ko}$	p=0.091	p=0.002	p=0.040
	$N^{wt/wt}/A^{ko/ko}$ vs $N^{ko/ko}/A^{wt/wt}$	p=0.098	p=0.049	p=0.014
	$N^{wt/wt}/A^{ko/ko}$ vs $N^{ko/ko}/A^{ko/ko}$	p=0.250	p<0.001	p=0.011
	$N^{ko/ko}/A^{wt/wt}$ vs $N^{ko/ko}/A^{ko/ko}$	p=0.707	p=0.442	p=1.000
14 DIV without PNNs	$N^{wt/wt}/A^{wt/wt}$ vs $N^{wt/wt}/A^{ko/ko}$	p=0.505	p=1.000	p=1.000
	$N^{wt/wt}/A^{wt/wt}$ vs $N^{ko/ko}/A^{wt/wt}$	p=0.002	p=0.002	p<0.001
	$N^{wt/wt}/A^{wt/wt}$ vs $N^{ko/ko}/A^{ko/ko}$	p<0.001	p<0.001	p<0.001
	$N^{wt/wt}/A^{ko/ko}$ vs $N^{ko/ko}/A^{wt/wt}$	p=0.328	p=0.021	p<0.001
	$N^{wt/wt}/A^{ko/ko}$ vs $N^{ko/ko}/A^{ko/ko}$	p=0.143	p<0.001	p<0.001
	$N^{ko/ko}/A^{wt/wt}$ vs $N^{ko/ko}/A^{ko/ko}$	p=1.000	p=0.652	p=1.000
21 DIV with PNNs	$N^{wt/wt}/A^{wt/wt}$ vs $N^{wt/wt}/A^{ko/ko}$	p=1.000	p=1.000	p=1.000
	$N^{wt/wt}/A^{wt/wt}$ vs $N^{ko/ko}/A^{wt/wt}$	p=0.618	p=0.001	p=0.024
	$N^{wt/wt}/A^{wt/wt}$ vs $N^{ko/ko}/A^{ko/ko}$	p=0.008	p=0.001	p<0.001
	$N^{wt/wt}/A^{ko/ko}$ vs $N^{ko/ko}/A^{wt/wt}$	p=0.110	p=0.003	p=0.012
	$N^{wt/wt}/A^{ko/ko}$ vs $N^{ko/ko}/A^{ko/ko}$	p=0.001	p=0.003	p<0.001
	$N^{ko/ko}/A^{wt/wt}$ vs $N^{ko/ko}/A^{ko/ko}$	p=0.697	p=1.000	p=1.000
21 DIV without PNNs	$N^{wt/wt}/A^{wt/wt}$ vs $N^{wt/wt}/A^{ko/ko}$	p=0.596	p=0.765	p=0.482
	$N^{wt/wt}/A^{wt/wt}$ vs $N^{ko/ko}/A^{wt/wt}$	p=0.689	p=0.494	p=0.326
	$N^{wt/wt}/A^{wt/wt}$ vs $N^{ko/ko}/A^{ko/ko}$	p=0.183	p=0.232	p=0.125
	$N^{wt/wt}/A^{ko/ko}$ vs $N^{ko/ko}/A^{wt/wt}$	p=0.419	p=0.077	p=0.101
	$N^{wt/wt}/A^{ko/ko}$ vs $N^{ko/ko}/A^{ko/ko}$	p=0.366	p=0.800	p=0.499
	$N^{ko/ko}/A^{wt/wt}$ vs $N^{ko/ko}/A^{ko/ko}$	p=0.073	p=0.005	p=0.130

The different culture conditions were subjected to pairwise comparisons with regard to the % increase/decrease of synaptic puncta. Twenty PNN-positive (n=20) and twenty PNN-negative neurons (n=20) were recorded and quantified for each condition. In total five independent experimental repetitions (N=5) were performed. Non-significant differences with p-value > 0.05 are shaded in grey. The Kolomogorov-Smirnov test was performed, in order to analyze the distribution of the data sets. Most data sets were not normally distributed and therefore the Kruskal-Wallis test was used to determine the p-values. Values concerning PSD95 puncta were normally distributed, so a univariate variance analysis (ANOVA) followed by a Scheffé post hoc test was undertaken.

Supplementary Table S2a: The average number of inhibitory synaptic puncta ± SEM and their percentage increase/decrease ± SEM compared to the $N^{wt/wt}/A^{wt/wt}$ – control for neurons with and without PNNs after 14 and 21 DIV

Condition	Genotype of astrocytes and neurons	Number of synaptic puncta ± SEM		
		vGAT	gephyrin	colocalization
**14 DIV with PNNs**	$N^{wt/wt}/A^{wt/wt}$	3060.8 ± 194.4	3329.4 ± 155.1	1685.4 ± 111.4
		0.0 ± 6.4 %	0.0 ± 4.7 %	0.0 ± 6.6 %
	$N^{wt/wt}/A^{ko/ko}$	2395.1 ± 119.5	3228.9 ± 140.4	1449.0 ± 70.6
		-21.8 ± 3.9 %	-3.0 ± 4.2 %	-14.0 ± 4.2 %
	$N^{ko/ko}/A^{wt/wt}$	2565.5 ± 190.7	3370.4 ± 134.8	**1283.5 ± 77.4**
		-16.2 ± 6.2 %	1.2 ± 4.0 %	**-23.9 ± 4.6 %**
	$N^{ko/ko}/A^{ko/ko}$	2669.3 ± 218.8	**2625.2 ± 91.4**	**1276.7 ± 67.1**
		-12.8 ± 7.2 %	**-21.2 ± 2.8 %**	**-24.3 ± 4.0 %**
**14 DIV without PNNs**	$N^{wt/wt}/A^{wt/wt}$	3759.6 ± 165.0	3626.7 ± 132.1	1944.7 ± 77.6
		0.0 ± 4.4 %	0.0 ± 3.6 %	0.0 ± 4.0 %
	$N^{wt/wt}/A^{ko/ko}$	3089.5 ± 136.7	3807.7 ± 104.3	1747.9 ± 70.7
		-17.8 ± 3.6 %	5.0 ± 2.9 %	-10.1 ± 3.6 %
	$N^{ko/ko}/A^{wt/wt}$	**2741.2 ± 142.0**	3668.3 ± 125.0	**1322.3 ± 70.7**
		**-27.1 ± 3.8 %**	1.2 ± 3.5 %	**-32.0 ± 3.6 %**
	$N^{ko/ko}/A^{ko/ko}$	**2623.3 ± 150.6**	**3129.8 ± 75.5**	**1262.0 ± 63.1**
		**-30.2 ± 4.0 %**	**-13.7 ± 2.1 %**	**-35.1 ± 3.2 %**
**21 DIV with PNNs**	$N^{wt/wt}/A^{wt/wt}$	3201.0 ± 141.5	3841.2 ± 176.2	1892.5 ± 92.4
		0.0 ± 4.4 %	0.0 ± 4.6 %	0.0 ± 4.9 %
	$N^{wt/wt}/A^{ko/ko}$	**4784.6 ± 273.0**	4049.5 ± 150.7	**2478.7 ± 129.1**
		**49.5 ± 8.5 %**	5.4 ± 3.9 %	**31.0 ± 6.8 %**
	$N^{ko/ko}/A^{wt/wt}$	3311.6 ± 190.4	**2864.0 ± 124.4**	1733.6 ± 101.2
		3.5 ± 6.0 %	**-25.4 ± 3.2 %**	-8.4 ± 5.4 %
	$N^{ko/ko}/A^{ko/ko}$	2827.7 ± 128.4	**3067.0 ± 134.6**	1646.3 ± 74.9
		-11.7 ± 4.0 %	**-20.2 ± 3.5 %**	-13.0 ± 4.0 %
**21 DIV without**	$N^{wt/wt}/A^{wt/wt}$	3133.4 ± 168.6	3443.0 ± 123.5	1674.3 ± 71.3

**PNNs**		0.0 ± 5.4 %	0.0 ± 3.6 %	0.0 ± 4.3 %
	$N^{wt/wt}/A^{ko/ko}$	**3972.6 ± 173.3**	3329.4 ± 99.9	**2041.4 ± 80.2**
		**26.8 ± 5.5 %**	-3.3 ± 2.9 %	**21.9 ± 4.8 %**
	$N^{ko/ko}/A^{wt/wt}$	3283.9 ± 132.3	**2698.3 ± 95.0**	1714.2 ± 75.6
		4.8 ± 4.2 %	**-21.6 ± 2.8 %**	2.4 ± 4.5 %
	$N^{ko/ko}/A^{ko/ko}$	2734.7 ± 104.2	3072.1 ± 110.2	1543.3 ± 57.8
		-12.7 ± 3.3 %	-10.8 ± 3.2 %	-7.8 ± 3.5 %

Synaptic puncta were visualized using laser-scanning microscopy, the average number per neuron is given. The % increase/decrease of individual puncta in comparison to the control condition ($N^{wt/wt}/A^{wt/wt}$) was calculated with reference to the average number of puncta determined for the control. Significant differences are highlighted in bold character, the p-values are listed in Supplementary Table S2b (see below).

Supplementary Table S2b: Table lists the p-values related to the altered numbers of inhibitory synaptic puncta between the four conditions for neurons with and without PNNs after 14 and 21 DIV.

Condition	Genotypes	p-value vGAT	p-value gephyrin	p-value colocalization
14 DIV with PNNs	$N^{wt/wt}/A^{wt/wt}$ vs $N^{wt/wt}/A^{ko/ko}$	p=0.052	p=1.000	p=1.000
	$N^{wt/wt}/A^{wt/wt}$ vs $N^{ko/ko}/A^{wt/wt}$	p=0.028	p=1.000	p=0.007
	$N^{wt/wt}/A^{wt/wt}$ vs $N^{ko/ko}/A^{ko/ko}$	p=0.062	p=0.008	p=0.016
	$N^{wt/wt}/A^{ko/ko}$ vs $N^{ko/ko}/A^{wt/wt}$	p=1.000	p=1.000	p=0.178
	$N^{wt/wt}/A^{ko/ko}$ vs $N^{ko/ko}/A^{ko/ko}$	p=1.000	p=0.017	p=0.321
	$N^{ko/ko}/A^{wt/wt}$ vs $N^{ko/ko}/A^{ko/ko}$	p=1.000	p<0.001	p=1.000
14 DIV without PNNs	$N^{wt/wt}/A^{wt/wt}$ vs $N^{wt/wt}/A^{ko/ko}$	p=0.035	p=0.372	p=0.667
	$N^{wt/wt}/A^{wt/wt}$ vs $N^{ko/ko}/A^{wt/wt}$	p<0.001	p=1.000	p<0.001
	$N^{wt/wt}/A^{wt/wt}$ vs $N^{ko/ko}/A^{ko/ko}$	p<0.001	p=0.049	p<0.001
	$N^{wt/wt}/A^{ko/ko}$ vs $N^{ko/ko}/A^{wt/wt}$	p=0.721	p=1.000	p<0.001
	$N^{wt/wt}/A^{ko/ko}$ vs $N^{ko/ko}/A^{ko/ko}$	p=0.108	p<0.001	p<0.001
	$N^{ko/ko}/A^{wt/wt}$ vs $N^{ko/ko}/A^{ko/ko}$	p=1.000	p=0.004	p=1.000
21 DIV with PNNs	$N^{wt/wt}/A^{wt/wt}$ vs $N^{wt/wt}/A^{ko/ko}$	p<0.001	p=0.807	p=0.005
	$N^{wt/wt}/A^{wt/wt}$ vs $N^{ko/ko}/A^{wt/wt}$	p=1.000	p<0.001	p=0.396
	$N^{wt/wt}/A^{wt/wt}$ vs $N^{ko/ko}/A^{ko/ko}$	p=0.287	p=0.004	p=0.354
	$N^{wt/wt}/A^{ko/ko}$ vs $N^{ko/ko}/A^{wt/wt}$	p<0.001	p<0.001	p<0.001
	$N^{wt/wt}/A^{ko/ko}$ vs $N^{ko/ko}/A^{ko/ko}$	p<0.001	p<0.001	p<0.001
	$N^{ko/ko}/A^{wt/wt}$ vs $N^{ko/ko}/A^{ko/ko}$	p=0.887	p=1.000	p=1.000
21 DIV without PNNs	$N^{wt/wt}/A^{wt/wt}$ vs $N^{wt/wt}/A^{ko/ko}$	p<0.001	p=1.000	p=0.007
	$N^{wt/wt}/A^{wt/wt}$ vs $N^{ko/ko}/A^{wt/wt}$	p=0.795	p<0.001	p=1.000
	$N^{wt/wt}/A^{wt/wt}$ vs $N^{ko/ko}/A^{ko/ko}$	p=0.879	p=0.098	p=1.000
	$N^{wt/wt}/A^{ko/ko}$ vs $N^{ko/ko}/A^{wt/wt}$	p=0.071	p<0.001	p=0.013
	$N^{wt/wt}/A^{ko/ko}$ vs $N^{ko/ko}/A^{ko/ko}$	p<0.001	p=0.193	p<0.001
	$N^{ko/ko}/A^{wt/wt}$ vs $N^{ko/ko}/A^{ko/ko}$	p=0.019	p=0.206	p=0.773

The different culture conditions were subjected to pairwise comparisons with regard to the % increase/decrease of synaptic puncta. Twenty PNN-positive (n=20) and twenty PNN-negative neurons (n=20) were quantified for each condition. In total five independent experimental repetitions (N=5) were performed. Non-significant differences with p-value > 0.05 are shaded in grey. Here, the Kolmogorov-Smirnov test was also performed to analyse the distribution of the data sets. All data sets were not normally distributed and therefore the Kruskal-Wallis test was used to determine the p-values.

Supplementary Table S3a: Mean values ± SEM of different parameters of the MEA analysis for all four conditions after 14 and 21 DIV

Parameter	Genotype of astrocytes and neurons	14 DIV	21 DIV
**Number of spikes**	$N^{wt/wt}/A^{wt/wt}$	2348.4 ± 153.3	4367.7 ± 261.3
	$N^{wt/wt}/A^{ko/ko}$	**3211.3 ± 176.6**	**3220.1 ± 210.3**
	$N^{ko/ko}/A^{wt/wt}$	1986.7 ± 129.5	5033.6 ± 360.7
	$N^{ko/ko}/A^{ko/ko}$	**4306.8 ± 217.9**	**8762.2 ± 455.0**
**Number of bursts**	$N^{wt/wt}/A^{wt/wt}$	57.6 ± 3.2	109.4 ± 5.8
	$N^{wt/wt}/A^{ko/ko}$	58.8 ± 2.7	**71.3 ± 4.3**
	$N^{ko/ko}/A^{wt/wt}$	**30.8 ± 3.0**	129.4 ± 8.3
	$N^{ko/ko}/A^{ko/ko}$	**101.0 ± 4.8**	**195.5 ± 6.8**
**Spike frequency [Hz]**	$N^{wt/wt}/A^{wt/wt}$	3.9 ± 0.2	7.3 ± 0.4
	$N^{wt/wt}/A^{ko/ko}$	**5.4 ± 0.3**	**5.4 ± 0.4**
	$N^{ko/ko}/A^{wt/wt}$	3.3 ± 0.2	8.4 ± 0.6
	$N^{ko/ko}/A^{ko/ko}$	**7.2 ± 0.3**	**14.6 ± 0.7**
**Spike frequency in burst [Hz]**	$N^{wt/wt}/A^{wt/wt}$	66.6 ± 2.4	86.2 ± 3.0
	$N^{wt/wt}/A^{ko/ko}$	**103.3 ± 3.9**	101.7 ± 4.4
	$N^{ko/ko}/A^{wt/wt}$	**48.5 ± 1.4**	75.2 ± 1.9
	$N^{ko/ko}/A^{ko/ko}$	**92.2 ± 4.2**	**94.3 ± 3.0**
**Percentage of spikes in burst [%]**	$N^{wt/wt}/A^{wt/wt}$	43.8 ± 2.2	50.4 ± 2.0
	$N^{wt/wt}/A^{ko/ko}$	**57.7 ± 2.2**	43.4 ± 2.4
	$N^{ko/ko}/A^{wt/wt}$	**24.0 ± 1.3**	50.8 ± 1.9
	$N^{ko/ko}/A^{ko/ko}$	**60.6 ± 1.9**	**71.5 ± 1.9**
**Burst duration [ms]**	$N^{wt/wt}/A^{wt/wt}$	341.9 ± 19.6	284.9 ± 12.8
	$N^{wt/wt}/A^{ko/ko}$	347.0 ± 11.2	**323.2 ± 35.0**
	$N^{ko/ko}/A^{wt/wt}$	457.0 ± 26.4	**325.1 ± 10.4**
	$N^{ko/ko}/A^{ko/ko}$	295.0 ± 8.8	**341.7 ± 10.7**

The table summarizes the parameters measured by MEA analysis of hippocampal networks *in vitro* cultivated for 14 or 21 days. Significant differences compared to the control ($N^{wt/wt}/A^{wt/wt}$) are highlighted in bold character. The p-values of the pairwise comparisons are detailed in Supplementary Table S3b (see below).

Supplementary Table S3b: p-values related to different parameters of the MEA analysis between the four conditions after 14 and 21 DIV

Parameter	Genotypes	p-value 14 DIV	p-value 21 DIV
**Number of spikes**	$N^{wt/wt}/A^{wt/wt}$ vs $N^{wt/wt}/A^{ko/ko}$	p=0.009	p<0.001
	$N^{wt/wt}/A^{wt/wt}$ vs $N^{ko/ko}/A^{wt/wt}$	p=1.000	p=1.000
	$N^{wt/wt}/A^{wt/wt}$ vs $N^{ko/ko}/A^{ko/ko}$	p<0.001	p<0.001
	$N^{wt/wt}/A^{ko/ko}$ vs $N^{ko/ko}/A^{wt/wt}$	p<0.001	p=0.010
	$N^{wt/wt}/A^{ko/ko}$ vs $N^{ko/ko}/A^{ko/ko}$	p=0.002	p<0.001
	$N^{ko/ko}/A^{wt/wt}$ vs $N^{ko/ko}/A^{ko/ko}$	p<0.001	p<0.001
**Number of bursts**	$N^{wt/wt}/A^{wt/wt}$ vs $N^{wt/wt}/A^{ko/ko}$	p=1.000	p<0.001
	$N^{wt/wt}/A^{wt/wt}$ vs $N^{ko/ko}/A^{wt/wt}$	p<0.001	p=1.000
	$N^{wt/wt}/A^{wt/wt}$ vs $N^{ko/ko}/A^{ko/ko}$	p<0.001	p<0.001
	$N^{wt/wt}/A^{ko/ko}$ vs $N^{ko/ko}/A^{wt/wt}$	p<0.001	p<0.001
	$N^{wt/wt}/A^{ko/ko}$ vs $N^{ko/ko}/A^{ko/ko}$	p<0.001	p<0.001
	$N^{ko/ko}/A^{wt/wt}$ vs $N^{ko/ko}/A^{ko/ko}$	p<0.001	p<0.001
**Spike frequency [Hz]**	$N^{wt/wt}/A^{wt/wt}$ vs $N^{wt/wt}/A^{ko/ko}$	p=0.016	p<0.001
	$N^{wt/wt}/A^{wt/wt}$ vs $N^{ko/ko}/A^{wt/wt}$	p=0.544	p=1.000
	$N^{wt/wt}/A^{wt/wt}$ vs $N^{ko/ko}/A^{ko/ko}$	p<0.001	p<0.001
	$N^{wt/wt}/A^{ko/ko}$ vs $N^{ko/ko}/A^{wt/wt}$	p<0.001	p=0.009
	$N^{wt/wt}/A^{ko/ko}$ vs $N^{ko/ko}/A^{ko/ko}$	p<0.001	p<0.001
	$N^{ko/ko}/A^{wt/wt}$ vs $N^{ko/ko}/A^{ko/ko}$	p<0.001	p<0.001
**Spike frequency in burst [Hz]**	$N^{wt/wt}/A^{wt/wt}$ vs $N^{wt/wt}/A^{ko/ko}$	p<0.001	p=0.136
	$N^{wt/wt}/A^{wt/wt}$ vs $N^{ko/ko}/A^{wt/wt}$	p<0.001	p=0.347
	$N^{wt/wt}/A^{wt/wt}$ vs $N^{ko/ko}/A^{ko/ko}$	p<0.001	p=0.031
	$N^{wt/wt}/A^{ko/ko}$ vs $N^{ko/ko}/A^{wt/wt}$	p<0.001	p=1.000
	$N^{wt/wt}/A^{ko/ko}$ vs $N^{ko/ko}/A^{ko/ko}$	p=1.000	p<0.001
	$N^{ko/ko}/A^{wt/wt}$ vs $N^{ko/ko}/A^{ko/ko}$	p<0.001	p<0.001
**Percentage of spikes in burst [%]**	$N^{wt/wt}/A^{wt/wt}$ vs $N^{wt/wt}/A^{ko/ko}$	p<0.001	p=0.920
	$N^{wt/wt}/A^{wt/wt}$ vs $N^{ko/ko}/A^{wt/wt}$	p<0.001	p=1.000
	$N^{wt/wt}/A^{wt/wt}$ vs $N^{ko/ko}/A^{ko/ko}$	p<0.001	p<0.001

	$N^{wt/wt}/A^{ko/ko}$ vs $N^{ko/ko}/A^{wt/wt}$	p<0.001	p=0.564
	$N^{wt/wt}/A^{ko/ko}$ vs $N^{ko/ko}/A^{ko/ko}$	p=0.445	p<0.001
	$N^{ko/ko}/A^{wt/wt}$ vs $N^{ko/ko}/A^{ko/ko}$	p<0.001	p<0.001
**Burst duration [ms]**	$N^{wt/wt}/A^{wt/wt}$ vs $N^{wt/wt}/A^{ko/ko}$	p=0.079	p=0.003
	$N^{wt/wt}/A^{wt/wt}$ vs $N^{ko/ko}/A^{wt/wt}$	p=0.032	p=0.006
	$N^{wt/wt}/A^{wt/wt}$ vs $N^{ko/ko}/A^{ko/ko}$	p=0.057	p<0.001
	$N^{wt/wt}/A^{ko/ko}$ vs $N^{ko/ko}/A^{wt/wt}$	p=0.443	p<0.001
	$N^{wt/wt}/A^{ko/ko}$ vs $N^{ko/ko}/A^{ko/ko}$	p=0.411	p<0.001
	$N^{ko/ko}/A^{wt/wt}$ vs $N^{ko/ko}/A^{ko/ko}$	p=0.384	p=0.124

The MEA analysis was carried out after 14 and 21 days for each of the four different culture conditions. The results were compared pairwise and the p-values for the different comparisons are given. Since the data were not normally distributed, the Kruskal Wallis test was used to identify significant differences. Five independent experiments (N=5) were performed and the data of all electrodes (n=60) were utilized for the statistics.

Supplementary Table S4a: Mean values ± SEM of the PNN area and its fluorescence intensity in the wildtype and quadruple knockout hippocampus *in vivo* at P15, P20, P25, P30 and P35

Parameter	Postnatal stages	Values ± SEM	
		WT	4xKO
**PNN area *in vivo***	P15	64155.5 ± 3273.7	**42558.5 ± 3223.2**
	P20	78005.2 ± 5021.1	**62810.4 ± 5409.4**
	P25	90590.7 ± 6056.0	**63750.3 ± 3300.5**
	P30	104404.1 ± 7588.3	**63620.7 ± 5082.0**
	P35	90108.0 ± 6146.0	73468.0 ± 8223.6
**PNN fluorescence intensity *in sections***	P15	4380059.0 ± 403847.2	**1963962.4 ± 150734.8**
	P20	5805772.8 ± 691451.3	**2820496.5 ± 436309.2**
	P25	5328356.2 ± 702004.8	**2299008.9 ± 160585.5**
	P30	7300182.0 ± 987441.8	**3305522.5 ± 395786.8**
	P35	5529794.88 ± 430019.6	4217469.63 ± 647443.4

PNN-containing areas in the CA2 region of the hippocampus as outlined in figure 6 were measured. Significant differences of the mutant as compared to the wild type tissue are highlighted in bold character. Furthermore, the fluorescence intensities were determined and compared. The corresponding p-values are specified in Supplementary Table S4b (see below).

Supplementary Table S4b: p-values related to the PNN area and fluorescence intensity comparing the wild type and quadruple knockout mouse hippocampus *in vivo* at P15, P20, P25, P30 and P35

Parameter	Postnatal stages	p-values WT vs 4xKO
**PNN area *in vivo***	P15	p<0.001
	P20	p=0.050
	P25	p<0.001
	P30	p<0.001
	P35	p=0.120
**PNN fluorescence intensity *in vitro***	P15	p<0.001
	P20	p=0.0018
	P25	p=0.0012
	P30	p=0.002
	P35	p=0.108

The mutant and wild type tissue sections were subjected to pairwise comparisons at corresponding age with regard to PNN covered surface area and fluorescence intensity. Three independent experiments (N=3) were used and four hippocampi (n=4) in two brain slices analysed for each condition. Non-significant differences with p-value > 0.05 are shaded in grey. The Kolmogorov-Smirnov test was performed for the determination of the normal distribution. For normally distributed data, the F-Test followed by an unpaired student’s t-test was chosen, whereas the Kruskal-Wallis test was utilized for the not normally distributed data.

Supplementary Table S5: Linear fold change of the gene expression within the P21 quadruple knockout hippocampus compared to the wildtype hippocampus and the p-value measured by micro array analysis

Genes	Linear fold change	p-value
Gabrq	1.52	p=0.0301
Cntnap4	1.50	p=0.0198
Gpc3	1.40	p=0.0089
Syt9	1.35	p=0.0029
Wnt7a	1.33	p=0.0002
Gad2	1.32	p=0.0062
Sema4c	1.16	p=0.0065
Calr	1.16	p=0.0038
Dnm3	1.11	p=0.0015
Sod3	1.10	p=0.0081
Cript	1.05	p=0.0083
Grin2d	-1.08	p=0.0057
Col4a3	-1.16	p=0.0014
Cplx3	-1.17	p=0.0037
Adamts13	-1.17	p=0.0058
Col27a1	-1.21	p=0.0098
Rapsn	-1.32	p=0.0010
Spon2	-1.33	p=0.0002
Col1a2	-1.35	p=0.0017

Linear fold change and significance levels are given for genes of interest.

Supplementary Methods S6

Cultivation of primary cortical astrocytes

Primary astrocytes were obtained from cortices of new born P1-3 mice following the established protocol by McCarthy et al. [1] with minor modifications. For co-cultivation of astrocytes and neurons, the astrocytes were plated out into cell culture inserts (Falcon by Thermo Fisher Scientific Inc.). Hence, the astrocytes were first washed with PBS before the enzymatic digestion with 3 ml 0.05 % (v/v) Trypsin-EDTA (T/E) (Gibco by Thermo Fisher Scientific Inc.; Cat. No.: 25300054) for 7-10 min at 37°C and 6 % CO_2 followed. The detached astrocytes were thereafter collected in 7 ml astrocyte medium and centrifuged at 216 g for 5 min. In a final step, the supernatant was carefully aspirated and the cell pellet was resuspended in 1 ml astrocyte medium. 25000 astrocytes in 500 µl culture medium were plated per insert. Two to four days after plating of the astrocytes into the inserts, the astrocytes should have grown to confluence on the insert membrane. After this period, the dissection of the hippocampi and the preparation of the neurons should be performed. In order to co-cultivate the astrocytes in the inserts with the neurons in particular 24-well plates, the medium used with the inserts was completely replaced by 500 µl serum-free hippocampus medium. Neurons have settled down 1 h after plating, thereafter the inserts were mounted into the particular 24-well plate, thereby allowing for an exchange of soluble factors through the permeable membrane. The co-culture of astrocytes and neurons was cultivated at 37°C and 6 % CO_2 in the incubator for periods of 14 and 21 DIV [2]. All inserts were placed into a conventional 24-well plate (Nunc by Thermo Fisher Scientific Inc.) with 500 µl astrocyte medium per well. The inserts were cultivated in the incubator at 37°C and 6 % CO_2.

After isolation of the cortices of overall three brains (≙ six cortices), the tissue was collected in 1 ml Hanks' Balanced Salt solution (HBSS) (-/-) (Gibco by Thermo Fisher Scientific Inc., Waltham, Massachusetts, USA, Cat. No.: 14170088). Enzymatic digestion was carried out for 15 min at 37°C using 1 ml Dulbecco′s Modified Eagle′s Medium (DMEM) (Thermo Fisher Scientific Inc.;Cat. No.: 41966029) containing 30 U/ml papain (Worthington Biochemical Corporation, Lakewood, New Jersey, USA; Cat. No.: LS003126), 0.96 µg/ml (w/v) L-cysteine (Sigma-Aldrich by Merck KGaA, Darmstadt, Germany; Cat. No.: C2529) and 80 µg/ml DNase I (Worthington Biochemical Corporation; Cat. No.: LS002007). Through adding 1-3 ml astrocyte medium (DMEM, 10% (v/v) horse serum (Sigma-Aldrich by Merck KGaA; Cat. No.: S9135), 0.1 % (v/v) gentamicin (Sigma-Aldrich by Merck KGaA; Cat. No.: G1397)), the digestion was stopped and the tissue was then gently triturated. The cell suspension was centrifuged in the following at 216 g for 5 min and the resulting cell pellet was re-suspended in 1 ml of fresh astrocyte medium. For plating of the cells, a T75 flask (Sarstedt AG & Co., Nuembrecht, Germany) pre-coated with 10 µg/ml poly-D-lysine (PDL) (Sigma-Aldrich by Merck KGaA; Cat. No.: P0899) in MilliQ water was used. The cell suspension was added to 9 ml astrocyte medium and the flask was cultivated at 37°C and 6 % (v/v) CO_2 in the incubator. The medium was completely changed every three days.

After seven to eleven days the mixed glial culture was grown to confluence and the flask was placed on an orbital shaker at 250 rpm for 1 h. Afterwards, the medium was completely exchanged and the cells were again incubated for about 1 h in the incubator. Then, the lid of the culture flask was closed with parafilm and the culture was shaken overnight to get rid of microglia and oligodendrocyte precursor cells (OPCs). The medium was replaced with fresh astrocyte medium on the next day and 20 µM cytosine-1-β-D arabinofuranosid (Ara-C) (Sigma-Aldrich by Merck KGaA; Cat.

No.: C1768) was added to the cultures for 48-72 h to remove the remaining OPCs and microglia to obtain a pure astrocytic culture. Cells were incubated at 37°C and 6 % (v/v)

Cultivation of primary hippocampal neurons

Neurons were isolated from the hippocampus of E15.5 mice as previously described, with a few modifications [2-4]. After dissection, the hippocampi of one litter were collected in 1 ml dissection medium (HBSS (Gibco by Thermo Fisher Scientific Inc.; Cat. No.: 14170088), 0.6 % (w/v) glucose, Serva Electrophoresis GmbH, Heidelberg, Germany; Cat. No.: 22700), 10 mM 4-(2-hydroxyethyl)-1-piperazineethanesulfonic acid (HEPES) (Gibco by Thermo Fisher Scientific Inc.; Cat. No.: 15630080)).

To digest the hippocampi, 1 ml Minimum Essential Media (MEM) (Gibco by Thermo Fisher Scientific Inc.; Cat. No.: 31095029) containing 30 U/ml papain, 0.96 µg/ml (w/v) L-cysteine and 80 µg/ml DNase I was added and incubated for 15 min at 37°C. The digestion was stopped by aspirating the digestion solution manually with a pipette and by adding 1 ml hippocampus medium (MEM, 10 mM sodium pyruvate (Sigma-Aldrich by Merck KGaA; Cat. No.: S8636), 2 % (v/v) B27 (Gibco by Thermo Fisher Scientific Inc.; Cat. No.: 17504044), 1 mg/ml (w/v) ovalbumin (Sigma-Aldrich by Merck KGaA; Cat. No.: A7641), 0.05 mg/ml (w/v) gentamicin). Afterwards, the hippocampus medium was carefully aspirated and new medium was added, this cycle was repeated three times. Then, the hippocampi were gently triturated to generate a single cell suspension. 35000 neurons per well (in 500 µl hippocampus medium) were plated out in special 24-well plates (Falcon by Thermo Fisher Scientific Inc.). Each well contained a glass coverslip (Menzel by Thermo Fisher Scientific Inc.), which had been previously coated for 1 h with 15 µg/ml poly-L-ornithine in MilliQ water (Sigma-Aldrich by Merck KGaA; Cat. No.: P3655).

References

1 McCarthy, K. D. & de Vellis, J. Preparation of separate astroglial and oligodendroglial cell cultures from rat cerebral tissue. *J Cell Biol* **85**, 890-902 (1980).

2 Gottschling, C., Dzyubenko, E., Geissler, M. & Faissner, A. The Indirect Neuron-astrocyte Coculture Assay: An In Vitro Set-up for the Detailed Investigation of Neuron-glia Interactions. *J Vis Exp*, doi:10.3791/54757 (2016).

3 Banker, G. A. & Cowan, W. M. Rat hippocampal neurons in dispersed cell culture. *Brain research* **126**, 397-342 (1977).

4 Geissler M., G. C., Aguado A., Rauch U., Wetzel H.C., Hatt H., Faissner A. Primary hippocampal neurons, which lack four crucial extracellular matrix molecules, display abnormalities of synaptic structure and function and severe deficits in perineuronal net formation *The Journal of Neuroscience* **33**, 7742–7755 (2013).
